# Centrosomal and Non-Centrosomal Microtubule-Organizing Centers (MTOCs) in *Drosophila melanogaster*

**DOI:** 10.3390/cells7090121

**Published:** 2018-08-28

**Authors:** Marisa M. L. Tillery, Caitlyn Blake-Hedges, Yiming Zheng, Rebecca A. Buchwalter, Timothy L. Megraw

**Affiliations:** Department of Biomedical Sciences, Florida State University, 1115 West Call St., Tallahassee, FL 32306, USA; cb16j@my.fsu.edu (C.B.-H.); yiming.zheng@med.fsu.edu (Y.Z.); rebecca.buchwalter@med.fsu.edu (R.A.B.)

**Keywords:** centrosome, centriole, *Drosophila*, microtubule-organizing center (MTOC), non-centrosomal MTOC, ninein, patronin, γ-tubulin, microtubule

## Abstract

The centrosome is the best-understood microtubule-organizing center (MTOC) and is essential in particular cell types and at specific stages during *Drosophila* development. The centrosome is not required zygotically for mitosis or to achieve full animal development. Nevertheless, centrosomes are essential maternally during cleavage cycles in the early embryo, for male meiotic divisions, for efficient division of epithelial cells in the imaginal wing disc, and for cilium/flagellum assembly in sensory neurons and spermatozoa. Importantly, asymmetric and polarized division of stem cells is regulated by centrosomes and by the asymmetric regulation of their microtubule (MT) assembly activity. More recently, the components and functions of a variety of non-centrosomal microtubule-organizing centers (ncMTOCs) have begun to be elucidated. Throughout *Drosophila* development, a wide variety of unique ncMTOCs form in epithelial and non-epithelial cell types at an assortment of subcellular locations. Some of these cell types also utilize the centrosomal MTOC, while others rely exclusively on ncMTOCs. The impressive variety of ncMTOCs being discovered provides novel insight into the diverse functions of MTOCs in cells and tissues. This review highlights our current knowledge of the composition, assembly, and functional roles of centrosomal and non-centrosomal MTOCs in *Drosophila*.

## 1. Introduction

The cytoskeleton maintains the internal organization of eukaryotic cells, providing the infrastructure necessary for cell shape, movement, mechanotransduction, polarity, organelle positioning, and intracellular transport, as well as chromosome movement and cytokinesis during cell division [1]. Microtubules (MTs) play a leading role in the cytoskeleton, and as the demands of the cell for its cytoskeleton change across time, developmental program, and cell type, the dynamic polymeric rods of MTs are specially engineered to meet these various needs.

MTs possess inherent polarity consisting of a faster-growing plus end, where the majority of elongation occurs, and a slower-growing minus end, which is typically stabilized by interactions or anchorage with a variety of proteins [1,2,3,4,5]. Because the organization of MTs is adaptable to different circumstances, understanding the localization and activity of the structures capable of controlling the nucleation, stabilization, and anchoring of MTs is critical to appreciating the diverse roles MTs play within the cell [3]. Structures with these capabilities are known as MT-organizing centers (MTOCs). More broadly defined, an MTOC is a site where the minus ends of MTs are localized [2,3,4]. By far the most extensively investigated MTOC is the centrosome, although non-centrosomal MTOCs (ncMTOCs) are clearly essential in their diverse contexts. This review will examine the roles centrosomes, centrioles, centrosomal proteins, and ncMTOCs play in a variety of cellular functions, highlighting our current knowledge of the assembly, composition, and functions of MTOCs at different stages and cell types throughout *Drosophila* development.

## 2. The Centrosome

**Figure 1:**
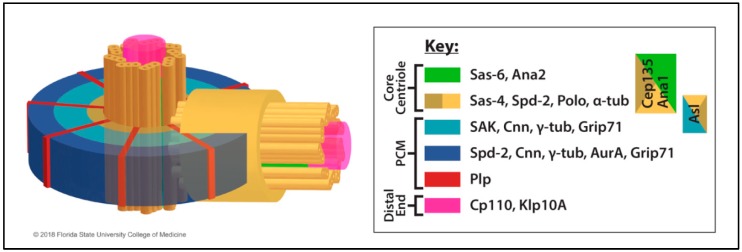
Structure of the *Drosophila* centrosome. The organization of several centriolar and pericentriolar material (PCM) proteins in the interphase centrosome. The mother centriole organizes PCM, shown as three layers, and maintains a tight association (engagement) with the daughter centriole. The figure is based on models presented in [16,17,18].

During interphase, the centrosome typically arranges MTs into a network suitable for intracellular transport [6,7,8,9,10]. During mitosis, the MT-assembly activity of the centrosome is significantly elevated, and a pair of centrosomes drives the organization of MTs into the bipolar spindle, an apparatus uniquely equipped to handle the faithful segregation of chromosomes to each daughter cell [2,11]. Consistent with the canonical centrosome structure in animal cells, the *Drosophila* centrosome typically consists of a pair of centrioles (a mother and a daughter) each composed of a nine-fold radially symmetric set of MTs (Figure 1). The length of the centrioles (see Section 2.2. Regulation of Centriole Length) and the number of centriolar MTs are dependent upon cell type and developmental stage [1,12,13,14]. A notable difference from vertebrate centrioles is the absence of distal and subdistal appendages on the mother centriole in *Drosophila* [15].

A matrix of proteins known as the pericentriolar material (PCM) assembles around the mature mother centriole. The structure of the PCM was long described as “amorphous” until superresolution microscopy enabled the visualization of a clear order of molecular components within the PCM [16,18,19] (Figure 1). The primary function of the PCM is the regulation of MTs. Although multiple regulators are involved in MT assembly, a key regulator of MTs at the PCM is γ-tubulin. Gamma-tubulin assembles into larger complexes including the γ-tubulin ring complex (γ-TuRC) consisting of γ-tubulin and γ-tubulin ring proteins (Grips) also known as γ-tubulin complex proteins (GCPs) (see further discussion in Section 3.1.1 Nucleator). 

Centrioles transition into basal bodies and, like their vertebrate counterparts, the mother centriole templates cilium assembly and also ciliary rootlet assembly in ciliated neurons [20]. The intraflagellar transport (IFT) machinery is essential for cilium assembly [21,22], whereas rootlets, which are necessary for cilium function, are not [23,24]. Although the fly does not need cilia during development, cilia are required for the sensory transduction of sensory neurons as well as for sperm function [21,22,25]. In spermatocytes, short cilia assemble on all four centrioles in the G2 phase and IFT is not required to form these cilia. Following meiosis, each spermatid inherits one centriole that will develop into a single spermatozoan flagellum [26,27,28]. Cilia will not be covered extensively, and the reader is referred to other reviews for more information [17,29].

### 2.1. Centriole Assembly

Having more than two centrosomes can result in multipolar spindles at mitosis, thereby increasing the likelihood of aneuploidy and genomic instability, both distinctive features of cancer [30,31]. Therefore, tight temporal control of centriole duplication is critical to ensuring that each dividing cell has only two centrosomes at mitosis. The core components involved in centriole assembly were discovered in *Caenorhabditis elegans* and are conserved in *Drosophila* [32,33]. Several recent reviews have covered centriole replication/biogenesis in depth [17,34,35].

**Figure 2:**
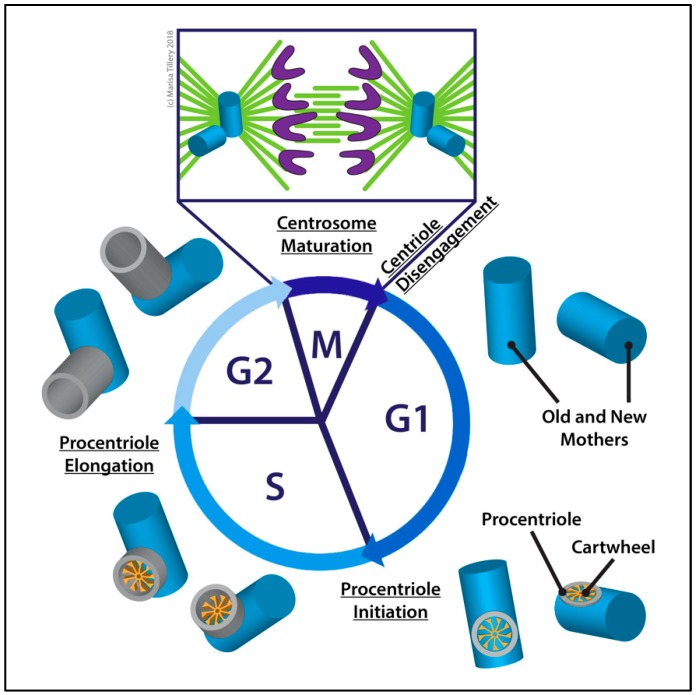
Centriole duplication cycle. Centriole pairs disengage in late mitosis, licensing mother and daughter centrioles to duplicate. Procentriole (grey) genesis begins with cartwheel (orange) assembly at the base of the old and new mother centrioles (blue) as cells enter S phase. Procentrioles elongate during S and G2 phases. During mitosis, DNA (purple) is segregated to each daughter cell through the activity of the centrosomes. PCM is recruited and the procentriole is converted to a mature mother capable of duplicating once the cell passes through mitosis. In late mitosis, the centriole pair disengages and the old and new mothers are licensed to duplicate.

Disengagement of each centriole pair in late mitosis (M phase) licenses them to duplicate in the next S phase [36] (Figure 2). Licensing of new daughter centrioles to duplicate for the first time in embryos requires *asterless* (*asl*) [37]. In *Drosophila*, disengagement involves the loss of Cyclin-dependent kinase 1 (Cdk1) activity but does not require cleavage by Cohesin as it does in vertebrates [38,39]. Prior to its inactivation in late mitosis, Cdk1 phosphorylates Spindle assembly abnormal 4 (Sas-4) at Thr200, creating a docking site for Polo kinase (Polo). The binding of Polo is required for both the recruitment of Asl to the daughter centriole and for centriole conversion, the process whereby the daughter centriole is converted to a mother centriole capable of duplicating and recruiting PCM to organize a centrosome [40]. Conversion requires the sequential loading of Centrosomal protein 135 kDa (Cep135/Bld10) [41], Anastral spindle 1 (Ana1) [42], and Asl onto the daughter centrioles during mitosis [43]. Immediately following the completion of mitosis, the new cell contains two centrioles, the old and new mothers, that will continue the cycle.

Following centriole disengagement, a nascent procentriole (the new daughter centriole) begins to form as a cartwheel on the proximal side of each G1 centriole. Spindle defective 2 (Spd-2), required for centriole duplication in other systems [44], is not required for centriole duplication in *Drosophila* except initially at fertilization [45,46]. Asl binds and recruits Polo-like kinase 4 (Plk4 or SAK) at its cryptic Polo box domain, leading to the localization and stabilization of Plk4 at the centriole at the onset of duplication [47,48,49]. Plk4 is the major regulator of centriole assembly in flies and other organisms [50,51]. The depletion of *plk4* by mutation or RNAi blocks centriole replication and therefore the formation of basal bodies [17,50,52], while its overexpression causes centriole amplification [47,48,50,53]. However, Plk4 recruitment to centrioles is not sufficient for centriole duplication in retinal cells [54]. Plk4 is downregulated by ubiquitin-mediated destruction via the Skp, Cullin, F box-small limbs (SCF-Slimb) complex [55,56,57]. Furthermore, its activation and regulation occur in an autologous trans-autoactivation fashion and require its restriction in space. This is critical to limiting centriole replication to one event per cycle [58]. Nevertheless, the precision by which centriole duplication is regulated has yet to be fully elucidated.

Plk4 binds and phosphorylates Ana2 [59], first at its N-terminal region and then at its C-terminal STil/ANa2 (STAN) domain, allowing it to recruit Sas-6 to the mother centriole to initiate cartwheel assembly [60,61,62]. Sas-6 proteins dimerize and then homooligomerize to form a nonameric ring from which their C-terminal domains radiate as spokes, generating the nine-fold symmetric cartwheel structure [17,63,64,65,66,67]. Cartwheel assembly is a critical early step in centriole biogenesis as it establishes the nine-fold radial symmetry of the centriole [61,68].

Sas-4 binds to Ana-2 and Cep135. Cep135 binds to Sas-6 at the outer rim of the cartwheel where Cep135 and Sas-4 likely contribute to centriolar MT assembly as they do in vertebrates [61,69,70,71,72,73]. Normal centriole duplication is exhibited by *cep135* mutant flies, but the cartwheel is less stable [41,74,75,76]. Cep135 is, however, required for centriole duplication in cultured *Drosophila* S2 cells [77]. Thus, Cep135 is required for duplication in some cell types but not in others [52].

Centriole cohesion, the tethering of the mother and daughter beginning at G1, does not seem to be a prominent feature in *Drosophila*. In cells such as neuroblasts and male germline stem cells where centriole dynamics have been extensively tracked, centrioles do not remain tethered. Consistent with this, Rootletin (Root), which is required in other systems for cohesion, is only expressed in sensory neurons where it maintains centriole cohesion [23].

Procentrioles grow in length through S and G2 phases but it is not until the cell passes through mitosis once again that the procentriole is converted to a mother centriole capable of recruiting PCM and replicating in the next duplication cycle.

### 2.2. Regulation of Centriole Length

The extreme diversity of centriole length in *Drosophila* depends on cell type, ranging from a short 0.18 µm in embryos to a very long 0.9 µm in spermatocytes [13]. How centriole length is controlled is not well-understood in *Drosophila* or in other organisms; however, clues to the regulation of centriole length come from mutations in several genes. Centriolar coiled-coil protein 110 kDa (CP110), a centriole-capping protein that restricts centriole elongation in mammalian cells [78], limits the elongation of centriolar MTs in vivo in *Drosophila* but is not required for centriole duplication, cell-cycle progression, or cilia/flagella assembly [79]. In S2 cells, on the other hand, *Cp110* knockdown results in shorter centrioles [80]. CP110 may regulate centriole duplication, as it restricts centriole amplification when *sas-6*, *ana-2* or *asl* are overexpressed [79]. CP110 interacts with Kinesin-like protein at 10A (Klp10A, a kinesin-13 family MT depolymerizer [81]). In *Klp10A* mutant cells or RNAi knockdown in S2 cells, centriolar MTs are longer, exhibit incomplete nine-fold symmetry, and are prone to break apart [17,79,80]. However, the effect of Klp10A on centriole length in S2 cells is not dependent on CP110 [80]. Mutants of another gene that restricts centriole length, *Basal body upregulated gene 22* (*Bug22*), exhibit longer centrioles and have difficulty assembling centriole pairs of proper shape and arrangement [17,82]. Additionally, Asl functions via Cep97 to regulate centriole length in somatic and germline cells, with *asl* mutants showing up to 30-fold elongation of centrioles [83].

Plk4, whose activity is autoregulated as mentioned earlier, regulates the rate of centriole elongation and also the period in which elongation occurs during S phase [84]. Other proteins that control centriole length include Cep135, Sas-4, Pericentrin-like protein (Plp), Proteome of centriole 1 (Poc1), and Ana1. In *cep135*, *sas-4*, *plp*, and *poc1* mutant spermatocytes, the centrioles and basal bodies are shorter than wild type [41,74,76,85]. The N-terminal region of Ana1, which interacts with Cep135 [43], promotes centriole elongation in a dose-dependent manner in *Drosophila* spermatocytes [42]. Once the mechanistic basis for centriole length control becomes more resolved, an understanding of the cell type-specific differences in centriole length can be approached.

### 2.3. Centrosome Pericentriolar Material (PCM) Assembly

As cells enter mitosis, the centrosome “matures” into a robust MTOC, a process that requires centriole conversion as described earlier (see Section 2.1. Centriole Assembly). Elucidation of the structural organization of both the interphase and the mitotic centrosome (Figure 1) was advanced by superresolution microscopy [16,18,19] and is covered in other reviews [17,34]. Centrosomin (Cnn) is a key regulator of PCM assembly [86,87,88,89], organizing the PCM scaffold and assembling outward from the centrioles [90,91]. Cnn recruits the γ-TuRC to the centrosome through the Centrosomin Motif 1 (CM1) domain of Cnn [92,93], and a mutation in the CM1 domain fails to rescue the *cnn* null mutant maternal effect embryonic lethality with associated defects in centrosome separation despite assembly of the PCM and formation of detectable MT asters [93]. Cnn is very dynamic in its assembly at centrosomes, including its organization of PCM “flares” that eject from centrosomes in a cell cycle-and MT-dependent flux [94].

Spd-2, Asl, Polo, and Plp are also involved in PCM assembly [47,95,96,97,98]. In *cnn* mutant neuroblasts, residual MTOC activity at mitotic centrosomes is abolished by mutation of *spd-2*, indicating some redundancy between Cnn and Spd-2 for PCM assembly [91]. Asl is required to recruit Cnn and Spd-2 to embryonic centrioles, and Cnn maintains Spd-2 at the PCM while Spd-2 promotes additional Cnn assembly [91]. Polo regulates centrosome maturation [99,100], and one of its key functions in this process appears to be its regulation of Cnn recruitment to centrioles [16,77]. Polo phosphorylates Cnn at mitotic centrosomes within its phosphoregulated-multimerization (PReM) domain to initiate PCM maturation [90]. Plp associates with the CM2 domain of Cnn and is required for efficient organization of the interphase PCM in cleavage-stage embryos [101,102]. Loss of Plp disrupts PCM structure similar to that seen in embryos carrying the *cnn^B4^* mutation in the CM2 domain [103]. Plp localizes to centrioles, the outer PCM, and also Cnn-containing flares [101,102] and is required for the organization of “PCM clouds” near the centriole wall at interphase centrosomes that appear to organize the PCM at mother centrioles [85].

### 2.4. The Centrosome Is ‘Dispensible’ for Mitosis

The perceived essential role of the centrosome in mitosis was challenged by the generation of an immortal *Drosophila* cell line 1182-4 that lacks centrioles [104] together with the discovery that female meiosis during oogenesis normally proceeds without centrioles [105,106,107,108,109,110]. Furthermore, the majority of zygotic development can occur efficiently with dysfunctional centrosomes or without centrosomes entirely as is the case in *cnn* [88] and *sas-4* [111] mutants, respectively. The ability to successfully accomplish cell division without centrosomes is not unique to *Drosophila*, however. Cell division in some planarians [112] and plants [113], as well as oocyte meiosis in the majority of animal species [114,115,116,117], routinely occurs without centrosomes [1]. Although centrosomes provide the dominant mechanism for MT organization into a bipolar spindle in *Xenopus* extracts, they are not required for spindle assembly as MTs are organized around mitotic chromatin when centrosomes are absent [116]. Consistent with this, unfertilized embryos in the gnat *Sciara coprophila* are able to initiate parthenogenic development and early mitotic cycles despite their lack of centrosomes [118]. Acentrosomal mechanisms for mitosis extend to mammalian cell culture as well. When the centrosome has been laser-ablated [119] or microsurgically removed [120], cells assemble functional bipolar spindles and complete mitosis [121]. Overall, these data indicate that an efficient spindle assembly pathway independent of centrosome activity exists in both vertebrate and invertebrate somatic cells. 

During acentrosomal spindle assembly, the intense pair of centrosomal asters that assemble in prophase are absent. Instead, MT bundles grow from the condensed chromosomes once the nuclear envelope breaks down [88,111,117,122,123,124]. The augmin complex is essential during acentrosomal spindle assembly, in part due to its involvement in kinetochore MT assembly [125,126,127,128,129]. In *Xenopus* oocyte extracts, chromatin-driven mitotic spindle assembly requires Ran-GTP [130,131,132,133] and spindle assembly factors TPX2 and HURP [126]; however, in *Drosophila* S2 cells, the Ran pathway is not required [134], but the chromosomal passenger complex (CPC) [135] is as in other systems [134,136]. In a screen for genes required for acentrosomal spindle assembly in S2 cells, several factors including the γ-TuRC were found to be required for spindle pole focusing [134]. Ultimately, acentrosomal cells efficiently assemble a bipolar spindle and execute mitosis effectively although these cells are more susceptible to mitotic delay, DNA damage, and death [11,34,111,137]. Thus, centrosomes are important in vivo for cell division in *Drosophila*.

In the absence of functional centrosomes, other MT-assembly regulators such as the augmin complex and other proteins that regulate MT assembly from kinetochores [126,127,129,137,138,139] become conditionally essential, and mutations in these genes enhance the phenotypes of centrosome protein mutants including synthetic lethality. Mitotic chromosome segregation appears to be more error-prone in centrosome protein mutants because *cnn*, *sas-4*, and *asl* mutants are lethal in combination with otherwise viable mutations in spindle assembly checkpoint mutants, with double mutants resulting in polyploidy and cell death [140,141].

### 2.5. Female Meiotic Spindle Assembly Is Acentriolar

A significant hallmark of female meiosis is that it occurs naturally in the absence of centrosomes, not only in *Drosophila* but in many other organisms as well. In these cases, the chromosomes serve as MTOCs [117]. RanGTP, essential for chromatin-mediated spindle assembly in other systems, is not essential for meiotic spindle assembly in *Drosophila* [142]. It is, however, required for efficient spindle pole focusing and also for sperm aster formation. Thus, RanGTP is important for MT assembly processes in the oocyte and early embryo, but not necessarily to generate the gradient at chromosomes for spindle assembly [142]. Mei-38, the Tpx-2 ortholog, is typically a spindle assembly factor regulated by RanGTP. It is required for chromosome arrangement on meiotic spindles and is proposed to support kinetochore-MT attachments [143].

*γ-tubulin at 37C* (*γTub37C*) is important for meiosis I spindle assembly and it encodes the maternal isoform of γ-tubulin, γTub37C, which localizes to meiotic spindle MTs [144]. The necessity of γTub37C for meiotic spindle formation has been controversial; some findings show it is necessary [109,144,145], while others found it is not because metaphase I spindles appear normal in *γTub37C* mutants [146,147]. These findings were reconciled in a careful study that examined meiosis I spindle assembly and dynamics live and with fixed specimens in *γTub37C* mutants [144]. In *γTub37C* mutant oocytes, prophase spindle formation and maintenance, chromosome alignment and kinetochore-MT attachments were impaired, and it was proposed that γTub37C may control MT nucleation at kinetochores [109,144]. However, consistent with findings from others, most spindles managed to recover and assemble into normal-looking metaphase I spindles despite chromosome orientation defects.

Non-claret disjunctional (Ncd) is a MT-bundling, minus end-directed kinesin-14 motor family member required for proper spindle assembly during female meiosis [106]. It plays a lesser role during mitosis [148], however, indicating its specific requirement for female meiotic acentrosomal spindle assembly. Ncd works cooperatively with other motor proteins [149] such as the MT-bundling, plus end-directed kinesin-5 family member Klp61F that slides antiparallel MTs [150] and further cooperates with the kinesin-12 Klp54D and the spindle pole protein Abnormal spindle (Asp) to establish meiotic spindle bipolarity [149]. Another kinesin, Klp10A, binds to the ends of MTs and promotes disassembly; reduced levels of Klp10A in oocytes causes elongated spindles and reduced EB1 turnover [151]. The MT-bundling, plus end-directed kinesin-6 motor family member Subito (Sub) is necessary for assembly and maintenance of the central spindle as well as the localization of the central spindle proteins of the CPC and the centralspindlin complex [145]. Altogether, a combination of MT-based motor proteins and MT-associated proteins coordinate acentrosomal spindle assembly in female meiosis.

Once spindle MTs are established, several proteins traffic to the spindle poles to maintain bipolarity and facilitate chromosome division. Centrosomal proteins are generally not found at the spindle poles [106,152,153] except for Transforming acidic coiled-coil protein (TACC) [154], a core centrosomal component that associates with the minus ends of spindle MTs, and its partner, the MT polymerase Mini spindles (Msps) [155,156]. Ncd transports Msps to the spindle poles where TACC anchors it, aiding the maintenance of spindle bipolarity [157]. The γTuRC may be involved in recruiting TACC to spindle poles since mutations in *γTub37C* or *grip71* (encoding a Nedd1 ortholog that interacts with the γ-TuRC and the augmin complex [139]) impair recruitment of TACC to the spindle poles [139,144]. However, co-immunoprecipitation assays did not reveal association of TACC with Grip71 or γTub37C and so the relationship between these proteins at the meiotic spindle poles is unclear [139].

The augmin complex recruits γ-tubulin to preexisting mitotic spindle MTs to nucleate MTs and increase spindle density [138]. It also plays a role during meiosis in chromosome congression [158]. In contrast to mitosis where the augmin complex is localized throughout the spindle and experiences a rapid turnover rate, during meiosis, it is stably present at the spindle poles through γ-tubulin and Ncd cooperation [158]. In *wac* (an augmin complex subunit) mutant oocytes, the MT density at the spindle poles is slightly reduced [127,158]. However, the bulk of spindle MT density is not perturbed, suggesting a separate role for the augmin complex during meiosis compared to mitosis.

#### Meiosis II Involves the Assembly of a Unique ncMTOC

**Figure 3:**
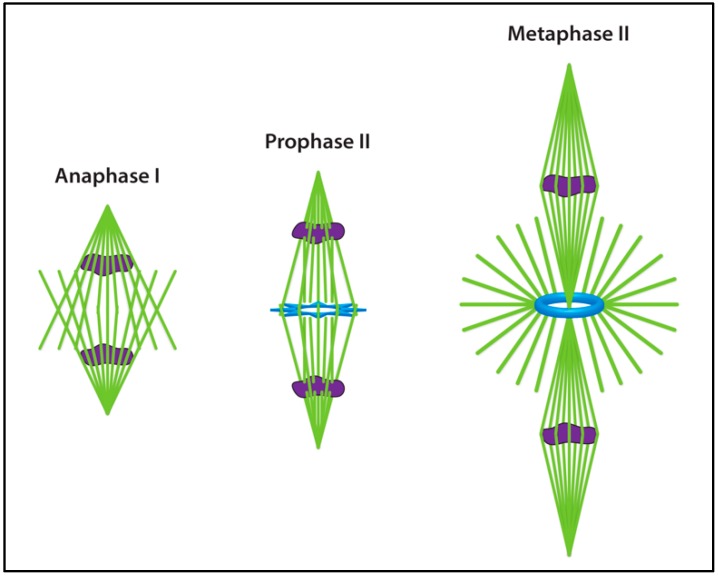
A non-centrosomal microtubule organizing center (ncMTOC) assembles during meiosis II. Meiosis I spindle assembly is anastral, but in late meiosis I a central aster forms, comprised of centrosomal PCM proteins, that separates the two meiosis II spindles. The blue structures represent the central aster ncMTOC; MTs are green; DNA is purple. Figure based on [152].

Once meiosis continues into anaphase I, a pucker of MTs forms within the central spindle, becoming the central aster of meiosis II in-between the two adjoined spindles [147,159] (Figure 3). The central aster acts as a central spindle pole for the two meiotic spindles of meiosis II, but it differs in composition from the outer spindle poles. The central aster is more similar to centrosomal spindle poles in that it produces astral MTs and contains centrosomal proteins such as γ-tubulin, Microtubule-associated protein 60 (Map60), CP190, Cnn, Mushroom body defect (Mud), and Asp, as well as the kinesin-6 motor Pavarotti (Pav) [160] better known for its role at the central spindle during cytokinesis [107,108,152,159,161,162,163]. However, the disk-shaped central aster ncMTOC is acentriolar.

The proteins necessary for formation of the central aster of meiosis II have come into focus. Mutations in *polo* [164], *cnn* [152], *mud* [163], or the γ-TuRC components *grip75* or *grip128* [165] block assembly of the central aster, resulting in separation of the two spindles at meiosis II and aberrant spindle organization. For a detailed review of *Drosophila* female meiosis see [166]. Successful completion of meiosis generates four haploid nuclei, one of which joins with the haploid sperm nucleus at fertilization.

### 2.6. The Centrosome Is Essential for Cleavage in Early Embryos

Late stage oocytes contain no centrioles but do contain the maternal components necessary to assemble functional zygotic centrosomes once centrioles have been contributed by the sperm at fertilization. The pair of centrioles contributed by the sperm will become the zygote’s first centrosomes [167]. If sperm nuclear reorganization is blocked, the sperm centrioles do not get activated and the embryo does not develop [168].

The sperm brings a pair of centrioles to the zygote: a “giant” centriole (GC) that previously functioned as the sperm basal body and a diminutive procentriole, also called the proximal centriole-like (PCL) [74,169]. Until recently, the PCL had been overlooked due to its small size, lack of centriolar MTs, and being “hidden” within the electron-dense centriolar adjunct where it assembles [74,169]. Centriolar proteins including Ana1, Poc1, Sas-6, and Cep135 localize to the PCL, which has a unique requirement for Poc1 for its assembly [74,167,170]. At fertilization, maternally-supplied PCM proteins such as Asl, Cnn, γ-tubulin, and Spd-2 are recruited to the GC and PCL in the early embryo [169]. Both the GC and PCL then replicate during the zygotic interphase to yield two functional centrosomes that assist in the first mitotic division [169,171]. The newly-created centrosomes assemble astral MTs that facilitate the migration of the female pronucleus farthest from the cortex towards the male pronucleus in a Ncd and Klp3A motors-dependent manner [164,171,172,173,174,175,176]. When male and female pronuclei fuse, the centrosomes separate to form the spindle poles of the first mitosis.

In the absence of centrioles, as in *asl* or *plk4* mutants or germline clones, or in embryos from *γTub37C* or *spd-2* mutant females, embryo development arrests with no divisions or, as in the case of the *sas-4* mutant, with less than eight spindles formed (indicating that fewer than four cleavage cycles have taken place) [45,96,162,177,178]. Spd-2 is critically required for PCM assembly at the GC and PCL, a role distinct from that in somatic cells where PCM assembly is impacted by loss of Spd-2 but not blocked [45,46]. In the absence of Spd-2 in the oocyte, astral MTs do not organize on the sperm centrioles and pronuclear fusion does not occur, resulting in embryonic arrest [45]. The unfertilized early *Drosophila* embryo, however, is competent to assemble centrioles de novo in the absence of sperm centrioles when *ana2*, *plk4*, or *sas-6* is overexpressed [53,59,179]. These de novo centrioles assemble PCM and astral MTs, but they cannot replicate [179]. 

#### 2.6.1. Cleavage Cycles

Early embryonic cleavage division cycles are rapid (averaging 8–12 minutes at 25 °C), synchronous, and syncytial (occurring in the absence of cytokinesis until cellularization of the blastoderm at cycle 14). By the end of 13 cleavage cycles, the embryo contains greater than 5000 nuclei residing in a shared cytoplasm at the cortex of the embryo. Although cortical nuclei are not surrounded by a cellular membrane, they manage to remain separate from one another through the activities of centrosomes and cytoskeletal proteins, especially during the last four cortical divisions [174].

**Figure 4:**
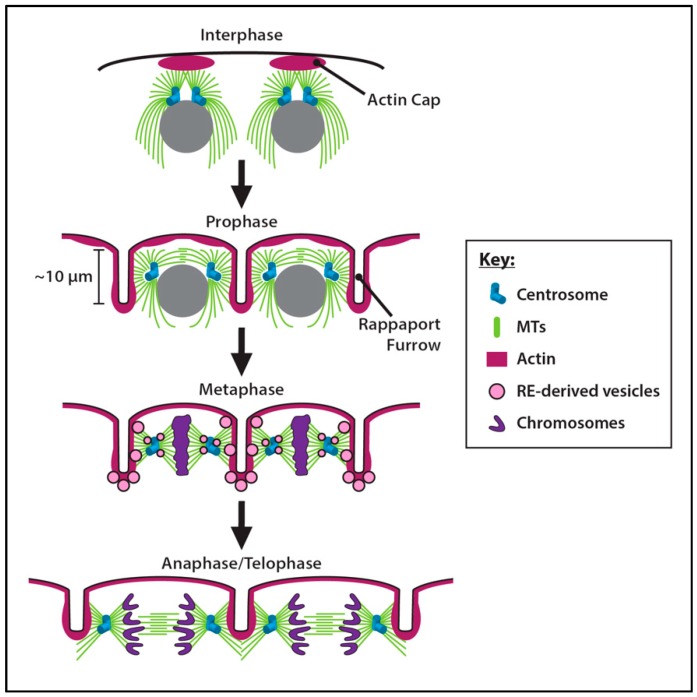
Centrosomes are essential to organize Rappaport furrows during embryonic cleavage cycles. The dynamics of centrosomes and furrow formation during cleavage cycles. Figure based on [174].

The first nine mitotic divisions occur deep within the embryo after which the nuclei, assisted by astral MTs emanating from the centrosomes, move to the cortex (cortical migration) [176,180] where the remaining divisions occur until cellularization. Once at the cortex, the apically-positioned centrosomes of each nucleus organize a cap of cortical actin below the plasma membrane (Figure 4, Interphase). Centrosomes are sufficient to reorganize actin at the cortex as centrosomes dissociated from their corresponding nuclei can direct cell cycle-dependent actin reorganization [181,182].

During prophase, centrosome pairs separate, and the actin caps spread into furrowed invaginations of the membrane (Figure 4, Prophase). Named for their similarity to the internuclear furrows first identified in sand dollar embryos [183], actin-membrane Rappaport furrows, also called cortical cleavage or pseudocleavage furrows, surround nuclei during embryonic syncytial blastoderm cleavage cycles in *Drosophila* and provide a physical barrier that prevents centrosomes from aberrantly interacting with the spindle apparatus of a neighboring nucleus [174,176,184]. The centrosomal protein Scrambled (Sced) and the Actin-related protein 2/3 (Arp2/3) actin nucleation complex are required for this expansion to occur [185,186].

In contrast to conventional cytokinetic furrows, these furrows require astral MTs but not spindle MTs. They form earlier than cytokinetic furrows and do not bisect the spindle [174,176]. However, Rappaport furrows and cytokinetic furrows contain nearly all the same actin cytoskeleton components [187], as well as the central spindle-associated proteins Polo and Fascetto (Feo) and the centralspindlin complex [188]. Although these components and the CPC are necessary for cytokinesis and are also present at the central spindle during cortical cleavage cycles, cytokinetic furrows are not formed over the central spindle [188]. These findings implicate a mechanism to block conventional cytokinetic furrow assembly in early embryos.

How Rappaport furrows are favored over cytokinetic furrows appears to involve the control of Rho GTPase by different classes of Rho GTP exchange factors (RhoGEFs). RhoGEFs activate Rho GTPases by catalyzing the exchange of GDP to GTP, stimulating their control of actin dynamics. During embryonic cleavage cycles, the RhoGEF Pebble (Pbl), which is necessary for cytokinesis and cellularization, is localized to neither the Rappaport furrow nor the central spindle. Rather, RhoGEF2 is present specifically at Rappaport furrows [188]. Introducing the active form of human Rho1 (RhoA) into cleavage-stage *Drosophila* embryos induces a cytokinetic furrow to form over the spindle [189]. This indicates that the spindle contains all components (apart from Pbl) that are necessary for conventional cytokinesis to otherwise occur and that RhoGEF2 activates Rho1 specifically at the Rappaport furrow as it is not necessary for cytokinesis at later stages [188,190].

The assembly of Rappaport furrows requires centrosomes and astral MTs. The centrosome plays the critical role of a transit hub during cleavage cycles for the MT-based trafficking of recycling endosome (RE)-derived vesicles that contain actin and plasma membrane components. These vesicles, regulated by Nuclear fallout (Nuf) and Rab11, traffic to the furrow to drive invagination (Figure 4, Metaphase) [191]. In many species, including *Drosophila*, Nuf acts as a Rab11-adaptor to dynein- and centrosome-associated REs. Both Nuf and Rab11 are necessary to recruit RhoGEF2 to furrows to support Rho1-dependent actin assembly [192] and failure to recruit Nuf and Rab11 to centrosomes impairs furrow formation. During cortical divisions, centrosomes from adjacent nuclei generate astral MTs that overlap at the site of membrane invagination forming Rappaport furrows (Figure 4, Anaphase/Telophase) [184]. Astral MTs are required during a critical window of the cleavage cycle. When MTs are disrupted by colchicine-injection in anaphase, furrows fail to assemble in prophase of the next cycle. Yet if colchicine is injected at telophase, furrows form in the next prophase. These data support the idea that the overlap of adjacent MT asters at anaphase are key to establishing the Rappaport furrow in the ensuing cleavage cycle [193].

Furrow formation is a critical function for centrosomes in embryonic cleavage cycles, as maternal *cnn* mutants assemble mitotic spindles and undergo nuclear divisions but fail to assemble Rappaport furrows, leading to highly erratic cleavage divisions and embryonic lethality [86,87,89,103]. TACC is required for astral MT assembly at embryonic centrosomes and is maternally-required for embryo survival [154]. TACC forms a complex with Msps, recruiting it to centrosomes [156]. AuroraA (AurA) kinase is required for TACC localization to centrosomes and an AurA-TACC-Msps complex regulates MT assembly at embryonic centrosomes through direct phosphorylation of TACC [194,195]. Loss-of-function *tacc* mutants are viable but maternal-effect lethal during cleavage stages where *tacc* is required early for pronuclear migration and then later for astral MT assembly in the minority of embryos that proceed into cleavage divisions [154].

Cnn is required for PCM assembly in cleavage stage embryos as it is in mitotic somatic cells [86,87,88,89,93,196]. Null mutations in *cnn* disrupt PCM assembly and are maternal-effect lethal due to failure to organize cleavage furrows [86,87]. In *cnn* mutant embryos, the mitotic spindles collide and fuse causing chromosomes to segregate aberrantly [86,87,93]. In embryos that express *cnn* with a mutation in the CM1 domain, γ-tubulin recruitment to centrosomes is reduced and centrosome separation is blocked, consistent with MT assembly impairment. However, some MT asters still form, and robust furrows are also assembled [93]. The CM2 domain targets Cnn to centrosomes, has *cis* and *trans* partners, and is necessary for mitotic centrosome assembly [197,198]. A point mutation in the CM2 domain that does not block centrosome localization but does disrupt PCM organization severely impairs cleavage furrow assembly; however, astral MTs are still prominent likely because the CM1 domain is intact [103]. Mutations that truncate the CM2 domain produce severe cleavage defects [86]. Centrocortin (Cen) is a partner of the CM2 domain that localizes to centrosomes and to Rappaport furrows. A mutation in *cen* disrupts Rappaport furrows, leading to spindle fusions [103]. Thus, Cnn supports Rappaport furrow assembly in embryos through MT assembly and also through the recruitment of Cen, a protein that supports furrow assembly through unknown mechanisms.

#### 2.6.2. Cellularization

The germ plasm, containing germ cell-specific mRNAs and proteins transported along centrosomal MT asters and deposited in oogenesis [199], is anchored at the posterior end of the embryo [200,201]. The few nuclei that reach the posterior pole and the germ plasm cellularize at cycle 10 before cellularization of the entire blastoderm at cycle 14. These nuclei become germ cell progenitors, also called pole cells. Experimentally separating nuclei from their centrosomes revealed that centrosomes are sufficient for cellularization of pole cells [181]. Centrosome separation and its regulation by *germ cell-less* (*gcl*) is necessary for centrosomes to coordinate pole cell formation [202].

During interphase of cycle 14, the remaining cortical nuclei undergo cellularization to form the multicellular blastoderm embryo. The apically-located centrosome pair associated with each nucleus forms a basally-extending basket of MTs allowing for nuclear elongation in the confines of the basket while the MTs grow to guide cellularization furrow growth. This process is driven by Golgi- and RE-derived vesicles, similar to the vesicles involved in furrow invagination [191]. The centrosomes also play a role in driving formation of these furrows as loss of astral MTs disrupts invagination [203,204]; however, centrosomes alone are not sufficient for blastoderm cellularization as they are for pole cells [181].

### 2.7. The Centrosome Is Essential for Cytokinesis in Spermatocytes

**Figure 5:**
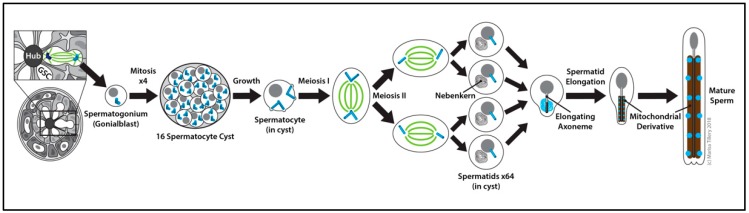
Spermatogenesis. Spermatogenic germline stem cells reside at a niche called the hub where divisions are polarized and asymmetric, producing spermatogonia. Spermatogonia divide four times to produce a cyst of 16 spermatocytes. Spermatocytes assemble short cilia from each of their four centrioles during G2 phase and retain them as they undergo two meiotic divisions to produce sixty-four haploid spermatids. Spermatids assemble an axoneme in their cytoplasm and the mitochondria fuse into two large mitochondrial derivatives upon which ncMTOCs assemble. Nuclei are grey; MTs are green; mitochondria are brown; MTOCs (centrosomes and ncMTOCs on the mitochondrial derivative) are blue.

The *Drosophila* testis supports the production and maturation of sperm throughout the life of the male fly (Figure 5). A small number of germline stem cells (GSCs) located in a specialized niche at the apical tip of the testis each divide asymmetrically every ~24 h to produce a renewed stem cell and a spermatogonial cell (also called a gonialblast) [205]. Proper orientation of the centrosome ensures that the orientation of the mitotic spindle is perpendicular to the niche and that the daughter cell is produced outside of it [206,207]. This is paramount to the successful production of differentiated cells and the maintenance of the stem cell pool. The centrosome orientation checkpoint involving Cnn and E-cadherin (E-cad) [207,208] monitors proper centrosome positioning and regulates cell-cycle progression [206,209]. Once this checkpoint is cleared, the spermatogonial cell undergoes four rounds of mitotic divisions with incomplete cytokinesis to create a cyst of 16 spermatocytes. It is critical to intercellular communication and the synchronization of differentiation that these spermatocytes remain interconnected via intercellular (cytoplasmic) bridges called ring canals [210,211,212]. The spermatocytes then experience a period of prolonged G2 phase growth in which the centrosomes dissociate from the nuclear membrane, move to the cell cortex, and assemble short cilia [26,27,28]. At meiosis, the two centriole pairs together with their short cilia migrate back toward the nucleus for spindle assembly [27,28]. The 16 diploid spermatocytes undergo two rounds of meiotic divisions to become 64 haploid spermatids each inheriting one centriole, which embeds into the spermatid nucleus and functions to template the basal body upon which the axoneme is assembled. In the early spermatid, a PCM-like structure called the centriolar adjunct assembles as a ring-like structure around the basal body within which a procentriole grows. In the mature spermatid, the centrosome is altered by losing many components of the PCM, a process known as centriole reduction [213]. Growth of the sperm axoneme proceeds from this complex and thus the final stage of spermatogenesis, mature sperm production, is achieved [212,214].

Although dispensable for successful divisions of GSCs and spermatogonia, the centrosome is essential for male fertility, being required for spermatocyte divisions [46,95,128,177,214,215,216,217,218]. Males with mutations in *asl*, *cnn*, or *plk4* are infertile and the testis show a characteristic defect in spermatocyte cytokinesis that manifests in the production of spermatids with multiple nuclei [50,169,214]. Despite severe impairment of spermatocyte cytokinesis, some spermatozoa form in *cnn* mutant testes, but they are immotile and exhibit defects in axonemal central pair MT formation, a likely cause of their motility defect. Furthermore, mutations in *γTub23C* or the γ-TuRC components *grip75*, *grip84*, *grip91*, or *grip 128* result in aberrant spindle assembly and defective cytokinesis [165,215,219,220], occasionally resulting in cleavage furrows that assemble but are positioned asymmetrically rather than over the central spindle [220]. This is consistent with the embryonic cleavage furrow requirement for Cnn, but it indicates that cytokinesis in spermatocytes is mechanistically distinct from other cell types in its unique requirement for centrosome function, perhaps relying on astral MTs for furrow regulation. Consistent with this reliance on centrosomal asters, cytokinesis in spermatocytes can proceed without chromosomes [221].

### 2.8. The Centrosome in Asymmetric Stem Cell Division

The population of cells that makes up an organism is diverse. Cell fate determination involves the asymmetric division of stem cells that commits a daughter cell to differentiation while simultaneously maintaining stem cell renewal. Organogenesis relies heavily on this mechanism. Asymmetric divisions have been described in the testis, nervous system, wing disc, muscle, gut, and Malpighian tubules [222,223]. Centrosome asymmetry and its roles in stem cell division have been well-characterized in male GSCs and neuroblasts [224].

#### 2.8.1. Male GSCs

**Figure 6:**
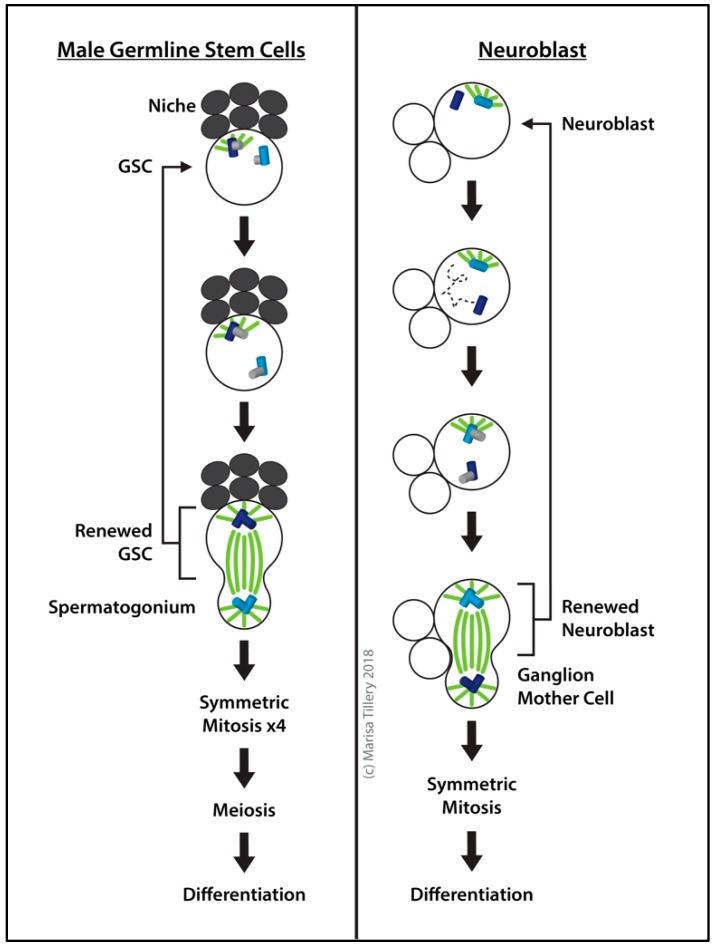
Male germline stem cells (GSCs) and neuroblasts exhibit centrosome asymmetry. Male germline stem cells retain the older mother centriole, which anchors at the apical membrane. Neuroblasts, on the other hand, anchor the daughter centrosome at the apical cell membrane. The neuroblast daughter centrosome maintains active MTOC activity during interphase, while the mother loses it until mitosis. Hub cells in the niche are dark grey; procentrioles are grey; centrioles with MTOC activity are blue: the daughter centrosome is light blue, the mother centrosome is dark blue.

Located at the apical end of the *Drosophila* male testis is a specialized stem cell niche, the hub, where GSCs undergo self-renewing asymmetric division to produce a spermatogonium destined for differentiation and a renewed GSC [225,226] (Figure 6). GSCs are attached to hub cells and surrounded by cyst cells that supply stemness signals. When GSCs divide, the stem cell remains at the hub while the daughter spermatogonium is delivered distally outside the niche [223].

The mitotic spindle is oriented perpendicular to the interface between the hub and GSC [227] (Figure 6). In contrast to neuroblasts (see Section 2.8.2. Neuroblasts), the GSC retains the older “mother” centrosome, which stays bound to the proximal cortex while the daughter centrosome is inherited by the spermatogonium [223,227,228,229]. The asymmetric inheritance of mother and daughter centrioles was demonstrated using the Pericentrin/AKAP450 centrosomal targeting (PACT) domain of Plp tagged with GFP under pulsed-inducible expression from a heat shock promoter [228,230]. With the mother anchored at the hub, the orientation of the GSC spindle at mitosis is established [228].

*cnn* loss-of-function mutants, which lack the astral MTs to anchor centrosomes to the cell cortex, cannot properly orient the mitotic spindle, and segregation of the mother and daughter centrioles is random [207]. In addition to Cnn, Adenomatous polyposis coli (Apc) 1 and 2 [231] are required for correct centrosome orientation by anchoring MTs [207]. Apc2 is enriched at the interface between the hub and GSC at the adherens junction, thus linking the adherens junction to providing a polarity cue that aids in the establishment of proper spindle and centrosome orientation [226]. Consistent with this role, ectopic expression of E-cad throughout the GSC cortex results in the mislocalization of Apc2 and an increase in misoriented centrosomes [208,232]. Centrosome disruption has no overt impact on asymmetric oogenic stem cell division [178]. In contrast, male GSCs arrest or delay the cell cycle until proper centrosome orientation is achieved [209]. The number of misoriented centrosomes in GSCs accumulates as the fly ages and it has been speculated that this contributes to a decline in spermatogenesis [227].

#### 2.8.2. Neuroblasts

*Drosophila* neuroblasts undergo self-renewing asymmetric division to produce a ganglion mother cell (that divides to produce neurons and glia) and a renewed neuroblast [233,234] (Figure 6). At interphase, the neuroblast centriole pair, surrounded by PCM and maintaining a robust MTOC near the apical cortex, splits apart. The daughter centriole remains at the apical cortex as an active MTOC while the mother centriole migrates away and rapidly loses its PCM [235,236,237,238]. Later, the mother centriole recruits PCM and regains MTOC activity during prophase of mitosis. Thus, in neuroblasts the centrosomes are asymmetric in PCM content and MTOC activity. Following division, the daughter centrosome remains with the renewed neuroblast, whereas the mother centrosome located near the basal cortex is inherited by the ganglion mother cell [235,236].

To divide asymmetrically, a neuroblast must correctly orient its mitotic spindle along the polarity axis. Astral MTs play a critical role in this through interactions with the cell cortex. Centrosome protein mutants lacking astral MTs such as *asl* [122,239], *cnn* [88], *sas-4* [111], *ana2* [240], or *centrobin* (*cnb*) [241] mutants fail to orient the mitotic spindle along the neuroblast polarity axis. Despite spindle alignment defects in mitosis, however, the majority of neuroblasts in these mutants correct their alignment and divide asymmetrically by telophase [88,111,122,239,241], indicating that compensatory mechanisms suppress the deleterious effects of centrosome loss on asymmetric stem cell divisions. When spindle orientation control is impaired or lost, symmetric, proliferative divisions occur more frequently, thus increasing the overall pool of stem cells [111,207,242]. Further evidence for the importance of centrosomal control of asymmetric division is the generation of tumors by explants of larval brains mutant for centrosome proteins or polarity genes [243].

Several proteins are involved in the recruitment and maintenance of PCM at the daughter centrosome. Cnb is localized at the daughter centriole and is necessary and sufficient for retaining MTOC activity. If Cnb is lost, the daughter behaves like the mother centriole and only matures at prophase of mitosis, and if Cnb is directed to both centrioles by expressing it as a Cnb-PACT fusion, it converts the mother centriole to a mature interphase centrosome like the daughter [241]. Plp, on the other hand, is more abundant at the mother centrosome and inhibits PCM retention there [235,244]. Cnn remains associated with the daughter centriole at the apical cortex with levels similar to mitotic centrosomes. The mother centriole, however, loses most if not all Cnn as it becomes inactivated [235,236,237,238]. The recruitment of Cnn and other PCM components to the interphase daughter centriole relies on the phosphorylation of Cnb by Polo [236,241]. Tryptophan-aspartic acid (WD) repeat domain 62 (Wdr62) stabilizes MTs to promote Polo recruitment to the interphase daughter centriole and PCM assembly [245]. The removal of Polo and the shedding of PCM from the mother centriole is blocked in *cep135* mutant neuroblasts [246]. Additionally, Partner of inscuteable (Pins) is required to activate the daughter centriole in interphase [237], however, the mechanism is unclear. Pins plays a role in spindle orientation and cell polarity [247,248,249,250] by binding directly to Mud, creating a Pins-G_αi_-Mud heterotrimeric complex. The association between Mud and Pins at the apical cortex is maintained by Ana2 and Cut up (Ctp), which work to regulate the function of Mud [240]. The Pins-G_αi_-Mud complex maintains localization of Inscuteable (Insc) and the Partitioning defective (Par) complex at the apical cortex of the neuroblast and aids in the regulation of spindle orientation [251,252,253].

## 3. ncMTOCs

The functions of centrosomes, the regulation of their assembly, and their control of MT assembly remains to be fully understood. Some cell types in *Drosophila* have no centrosomes or have inactive centrosomes. In these cells, ncMTOCs become critical organizers of the MT array [254]. Following loss or inactivation of the centrosome, differentiated cells often reassign MTOC function to a site that does not include the centrosome, generating alternative ncMTOCs that vary by cell type. While differentiation plays an important role in the switching between MTOCs, the assembly of many ncMTOCs, as well as their basic architecture, remains largely undiscovered. The transition from centrosomal to ncMTOCs during cell differentiation is poorly understood, but several models for this transition have been presented in several excellent reviews [2,3,254]. ncMTOCs function similarly to centrosomal MTOCs, but they also serve unique and critical functions to the cells that employ them [2,3,254]. MT organization at ncMTOCs is regulated by diverse mechanisms as highlighted in the individual examples described below.

### 3.1. Key Microtubule (MT) Minus End Regulators for ncMTOCs

ncMTOCs are diverse in their subcellular localizations and molecular composition and there is relatively little understanding of their assembly and functions in development and disease [2,3]. While the exact nature of ncMTOCs is beginning to come to light, a fundamental tenet is that ncMTOCs comprise proteins that associate with MT minus ends as well as proteins that act as adapters to couple MT minus end proteins to a subcellular locale. A model has been proposed wherein ncMTOCs employ MT nucleators, stabilizers, and/or anchors to fulfill their basic function of organizing MT minus ends. Gamma-tubulin, Patronin, and Ninein (Nin) have emerged with key roles as nucleator, stabilizer, and anchor of MTs, respectively [2].

#### 3.1.1. Nucleator

Gamma-tubulin was the first MT minus end protein identified [2,255]. As previously discussed, γ-tubulin plays a key role in MT nucleation [13], functioning in heteromultimeric complexes including the γ-TuSC and the γ-TuRC [256,257,258,259]. As in other eukaryotes, multiple γ-TuSCs associate with other Grip/GCP proteins to form the γ-TuRC in *Drosophila* [260], but the γ-TuSC is sufficient to associate with the centrosome and nucleate MTs [256,261]. At centrosomes γ-tubulin is a major, but not the sole, regulator of MTs.

The capacity of the γ-TuRC to act as a nucleator depends upon the adaptor that recruits it to the site of the MTOC [262]. The aforementioned augmin complex is a γ-TuRC regulator, facilitating MT assembly from preexisting MTs [263,264]. The CM1 domain from Cnn and its orthologs is a γ-TuRC activator [265] and is sufficient to generate an MTOC when anchored to a specific subcellular location [92,262,266]. In vertebrate cells, Nedd1 anchors the γ-TuRC without stimulating MT nucleation [262]. In *Drosophila*, Grip71, the Nedd1 ortholog, is required to recruit γ-tubulin to spindle MTs but not to centrosomes, yet a role at ncMTOCs has not been reported [139]. Mozart, a more recently-discovered component of the γ-TuRC, associates with the N-terminal regions of Grip proteins and may be an adaptor for the CM1 domain and Grip71 [259]. *Drosophila* encodes a single Mozart protein but its expression and function are restricted to late spermatogenesis [267]. A recently-discovered γ-TuRC adaptor and activator, Nme7 [268], is uncharacterized in *Drosophila*.

#### 3.1.2. Stabilizer

The Patronin/CAMSAP family of proteins are MT minus end-associated proteins [3,269] that stabilize MT minus ends by acting as caps to prevent depolymerization by kinesin-13 proteins [270]. This antagonism between Patronin and kinesin-13 was seen in S2 cells [269] and in oocytes [271]. Patronin has nucleator-like properties; however, it is possible that Patronin recognizes and caps MTs released from the γ-TuRC or MTs produced by the MT-severing enzyme katanin [2,272]. When *patronin* is depleted, the overall number of MTs decreases, MTs become less organized, and the number of free-moving MTs increases [2,269,270]. Recent findings indicate that Patronin is a key MT regulator at a variety of ncMTOCs, yet its mechanisms of action in vivo are not well understood.

#### 3.1.3. Anchor

One mechanism to generate an MTOC is to provide a MT anchoring site. Although Nin has not been shown to interact directly with MT minus ends, evidence points to it functioning as a MT anchor, notably in the ncMTOCs of various epithelial cell types such as murine cochlear cells [273], differentiated murine epidermal cells [274], murine myotube culture [275], and *C. elegans* epidermis [276]. Nin is a coiled-coil protein mutated in Seckel syndrome (SCKL, OMIM 210600) [277] that was first identified as a centriolar protein [278]. In mammals, Nin is localized to the centriole wall and the subdistal appendages on the mother centriole [279]. A developmental switch from a centriolar splice variant of Nin to a non-centriolar splice variant is essential for murine neural progenitor cell differentiation into neurons [280]. *Drosophila* centrioles, however, lack subdistal appendages [29] and so the function of Nin in *Drosophila* must be related to non-centriolar roles [281]. *Drosophila nin* is non-essential, but Nin is localized to the centrosome periphery in embryos and at least to some ncMTOCs. Its ectopic expression is lethal during development [281]. Whether Nin has an important function at all ncMTOCs in *Drosophila* awaits further investigation. Nin may not be the only anchor, though; in some contexts, it appears that γ-TuRCs can serve as MT anchors, perhaps using Grip71 as an adaptor as mentioned above.

The molecules mentioned here may not be the only MT minus end regulators or “effectors” of the MTOCs; several additional effectors are implicated in our discussion to follow. Further discovery of the full spectrum of MT minus end regulators will inform us on the diversity of ncMTOCs that serve the variety of functions in the cell types they support.

### 3.2. ncMTOCs in Somatic Cell Development

#### 3.2.1. Epithelial Tissues

Epithelial layers emerge early during animal development. Investigations of *Drosophila* epithelial tissues have proven invaluable for understanding the genes and mechanisms involved in cell fate determination, cell proliferation, differentiation, and morphogenesis. ncMTOCs enable execution of the functions of epithelial cells and also their morphogenesis into the organs they support.

##### Ovarian Follicle Cells

**Figure 7:**
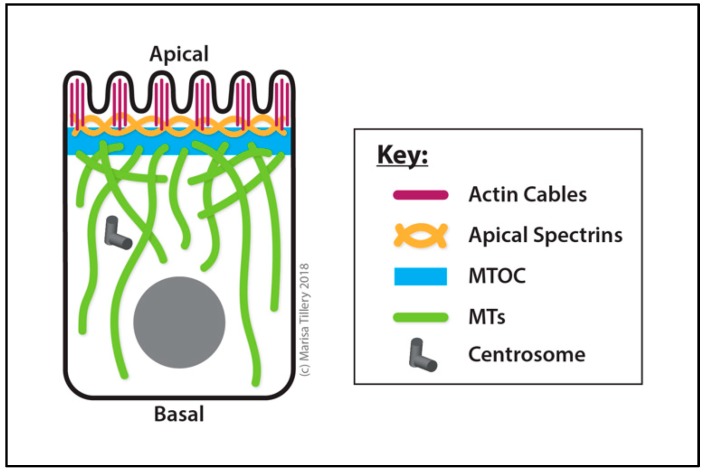
Organization of the ncMTOC in ovarian follicle cells. Shot, not depicted in the illustration, is associated with the apical spectrin cytoskeleton as is the ncMTOC.

During oogenesis, follicle cells form an epithelial monolayer that surrounds each egg chamber. These cells exchange important signaling cues to direct the polarity and development of the oocyte as well as their own morphogenesis [282,283]. Follicle cells assemble a ncMTOC at their apical membrane that regulates apical microvilli morphogenesis [271,284] (Figure 7). Disruption of the ncMTOC also affects nuclear positioning. This ncMTOC is required for the trafficking of the protocadherin Cadherin 99C (Cad99C) by Rab11 endosomes via dynein motor and its adaptor Nuf to regulate apical microvilli morphogenesis [284]. Disruption of the MT array either with colchicine treatment or by overexpression of *katanin* blocks apical recruitment of Cad99C and Rab11 vesicles.

The assembly of this apical ncMTOC requires Patronin, the spectraplakin Short stop (Shot, a MT-actin crosslinker) [285], and β_H2_-spectrin cooperatively. Shot and β_H2_-spectrin may function redundantly in Patronin recruitment to the apical membrane [284]. Mutational disruption of this ncMTOC impairs MT organization, Rab11 trafficking and Cad99C incorporation at the apical membrane. Like follicle cells, the oocyte (see Section 3.2.2. Oocyte: Transition from Centrosomal to ncMTOCs) has a membrane-anchored ncMTOC that requires Patronin and Shot [271], but unlike the follicle cell ncMTOC, it appears strictly dependent on Shot for its apical cortical localization.

##### Salivary Gland

The *Drosophila* salivary gland is an epithelial secretory tissue that forms during embryogenesis and is composed of two tubes made up of a monolayer of highly polyploid cells [286]. Early in its development, a group of epithelial cells transition from cuboidal to columnar in shape and form the salivary gland placode [287]. During early- to mid-stage 11 of embryogenesis, placode cells stop dividing but continue to increase in volume and ploidy and begin morphogenesis to form the salivary gland tubes. Cells located near the dorsal-posterior corner of the placode begin to constrict their apical surfaces leading to invagination of the early tube in mid- to late-stage 11 [288].

**Figure 8:**
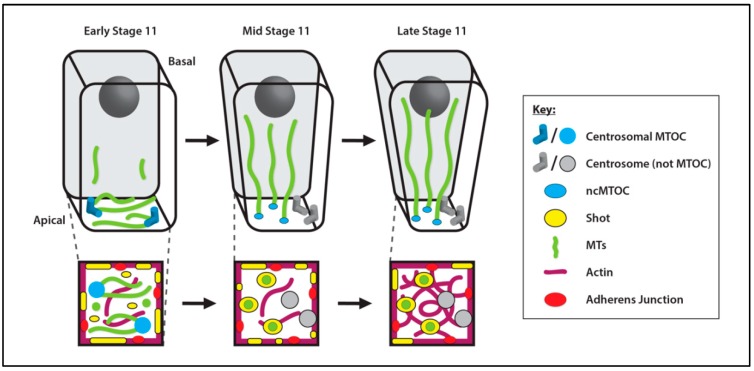
Salivary gland placode cell ncMTOC. In this cell type, the MTOC changes dynamically during embryonic stage 11 from a centrosomal MTOC to an apical membrane ncMTOC required for morphogenesis.

During early stage 11, MTs in placode cells are organized from centrosomes and oriented parallel to the apical plasma membrane surface (Figure 8). However, at mid stage 11, the MT cytoskeleton undergoes a dramatic rearrangement and MTs reorient by 90° to align parallel to the apicobasal axis (perpendicular to the apical plasma membrane surface) (Figure 8). These longitudinal MTs emanating from the apical surface are enriched in acetylated-tubulin, indicating that they are relatively stabilized, and they no longer localize to the centrosomes [288]. Expression of the motor fusion proteins No distributive disjunction (Nod)-β-galactosidase and kinesin-β-galactosidase, two well-established tools to mark MT minus and plus ends, respectively, [289], confirmed that MT minus ends are located at the apical membrane and that plus ends extend toward the basal side of the cell. Asl and γ-tubulin are components of this apical membrane ncMTOC, but their requirement has not yet been established. Thus, at the onset of morphogenesis into a tube, MT organization switches from centrosomal to an apical membrane ncMTOC [288].

Following the 90° reorientation of the MT cytoskeleton, MT bundles come into close contact with accumulations of apical actomyosin. The actomyosin network in placodal cells is highly dynamic, being responsible for the pulses of constriction that drive invagination and tube formation. If the interaction of MTs with this apical actomyosin network is lost, as is the case when *spastin* (*spas*, encoding a MT-severing enzyme) [290] is overexpressed or when a fusion of the MT-binding domains of Shot are overexpressed, apical constriction is hindered, impairing morphogenesis and salivary gland tubulogenesis [288].

##### Trachea

The *Drosophila* trachea is an elaborate, tubular epithelial network that satisfies the respiratory requirements of the fly. Air enters through spiracles along the sides of the body and travels through tubes of polarized epithelial cells where oxygen and CO_2_ exchange occurs [291]. Proper morphogenesis and branching of the tubular tracheal network are dependent upon anterior–posterior and dorsal–ventral signals and is mostly completed by the end of embryogenesis. This system serves as an excellent and important model for organogenesis of ductal tissues, or tubulogenesis [286].

**Figure 9:**
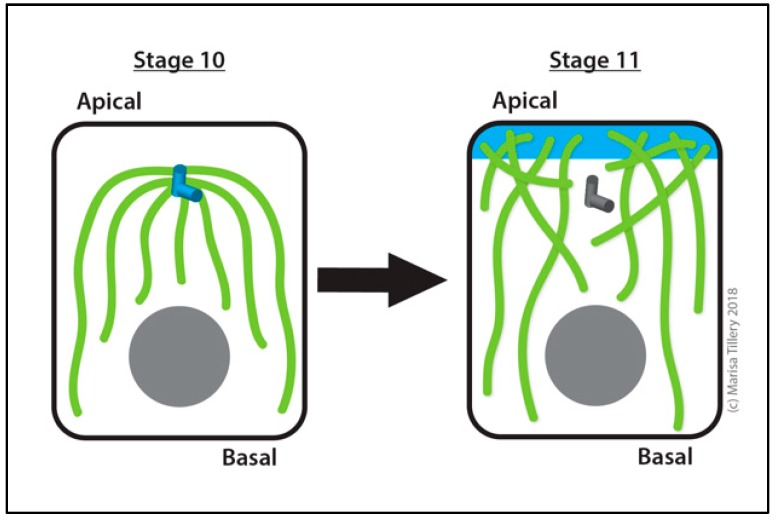
An apical membrane ncMTOC assembles in tracheal epithelia. The MTOC is blue and transitions from the centrosome to an apical ncMTOC; MTs are green; the nucleus is grey.

During embryonic development of the trachea, the MTOC activity of the centrosome is inactivated in invaginating epithelial cells (non-dividing cells that eventually morph into elaborate tracheal branches) and a ncMTOC located at the apical plasma membrane forms and dominates MT organization [292] (Figure 9). Differentiation signaling regulates the MTOC conversion as the switch does not occur in a *trachealess* (a transcription factor involved in tracheal differentiation) loss-of-function mutant [292,293,294]. These changes and their developmental timing appear analogous to the epithelial cells in the salivary gland placode. Coincident with this transition, the γ-TuRC shifts from localization at the centrosome to localization along the apical membrane in these cells and, following MT depolymerizing treatment, MTs regrow from the apical plasma membrane. Endogenous Spas first releases the γ-TuRC from the centrosome and then Piopio [295] (Pio, a membrane protein involved in tracheal branch growth) facilitates the attachment of the γ-TuRC to the apical membrane. Exactly how Spas regulates the proposed transfer of γ-TuRCs from the centrosome to the apical membrane and the role of Pio in MTOC assembly at apical epithelial membranes requires further investigation [292]. Based on their involvements in other epithelial ncMTOCs, additional candidates for contributors of function to this ncMTOC may include Shot and Patronin.

Blocking the formation of this ncMTOC during embryonic development affects secretion of chitin into the lumen and disrupts tracheal morphogenesis, producing trachea invagination and branching defects [292]. ncMTOC disruption was achieved by the overexpression of *spas*; cold treatment to disassemble MTs; RNAi depletion of *grip84*, *grip128*, or *grip163*; and with a *grip84* mutant. RNAi depletion of *grip128* or *grip163* (components of the γ-TuRC but not the γ-TuSC) results in loss of Grip84 recruitment to the ncMTOC in tracheal cells, indicating that the γ-TuRC is required for ncMTOC activity [261].

An apical ncMTOC in mammalian epithelial cells has also been observed and is important for establishing apical–basal cell polarity. This polarized arrangement of MTs provides a highway to effect MT-dependent cell trafficking [296]. The tracheal ncMTOC may similarly regulate protein trafficking as the MT array is essential to establish the apical supply of adherens junction proteins in later-stage embryonic tracheal cells located at a structure called the dorsal branch [297].

Tracheal terminal cells (TCs) lie at the distal tip of the developing tracheal branches. Although TCs are associated with the tracheal epithelium, they do not appear to form a ncMTOC. Instead, they employ centrosomes (normally two) localized near the basal membrane to generate a polarized MT array that facilitates assembly of an intracellular lumen. In later post-mitotic stages of embryonic development, the experimental amplification of centrosomes results in aberrant branching of TCs and the luminal extension into the TCs frequently bifurcates [298]. In addition, in *sas-4* mutant TCs, luminal extension formation is impaired. Therefore, in this system, centrosomal and non-centrosomal MTOCs are critical for development. 

##### Wing

**Figure 10:**
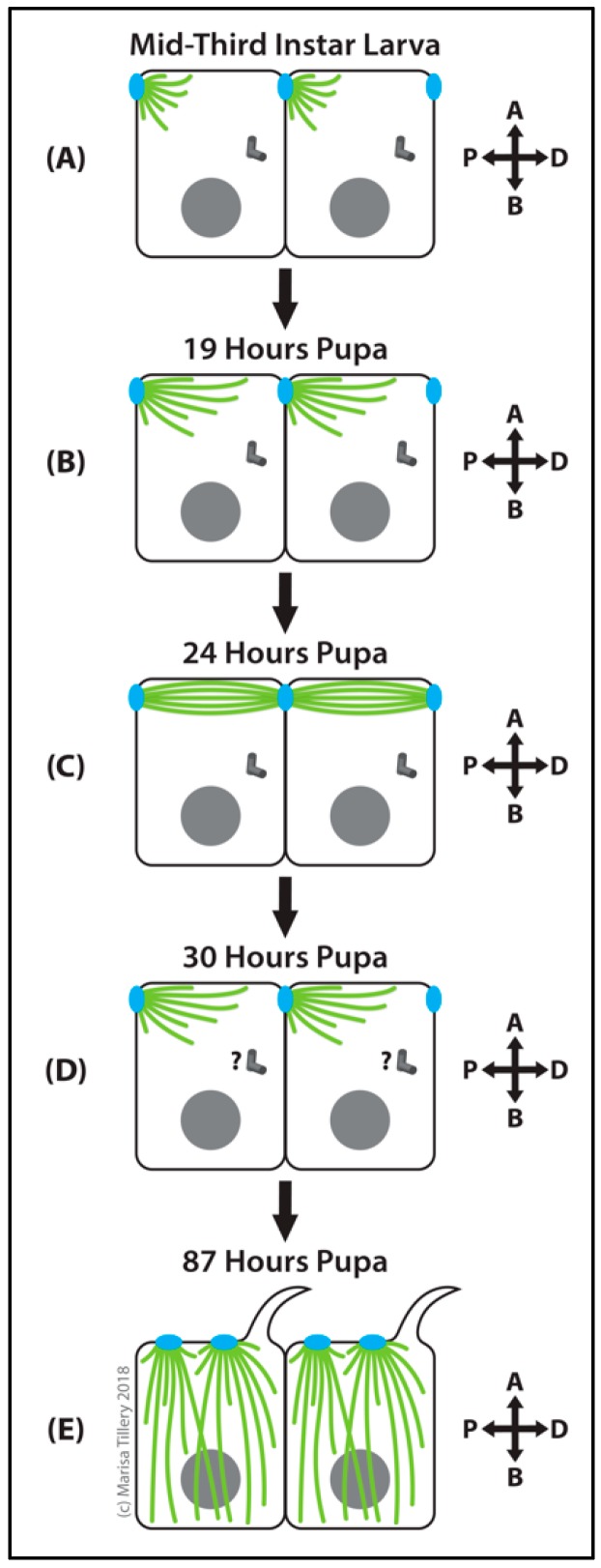
Wing epithelial cell ncMTOC. During larval and early pupal stages (**A**–**C**), wing epithelial cells employ an apical junction to assemble a dynamic ncMTOC along the proximal–distal axis that controls planar cell polarity. By 30 h into pupal development (**D**), the MT array loses its ordered proximal–distal organization. At the final stages of wing morphogenesis (**E**), a ncMTOC forms at the apical surface of trichome-bearing cells and organizes MT arrays along the apical–basal axis. The ncMTOC is blue; MTs are green; nuclei and centrosomes are grey.

Similar to other epithelial cell types, MTs in the *Drosophila* wing epithelia are polarized with apical anchorage of MT minus ends [299,300]. Although centrosomes are present near the apical sides of these cells, they are not the dominant sites of MT nucleation or anchoring. Instead, during the early- to mid-third larval stage, MTs first appear as dense, short, focused bundles that are asymmetrically-anchored to apical intercellular junctions at the proximal side of the cell [301] (Figure 10A). During early pupal wing development, apical junctions become the major ncMTOC [301,302,303]. These intercellular “spot” junctions are located on the lateral membrane near the apical surface on the proximal and distal sides of each columnar epithelial wing cell (Figure 10). In the early pupal wing epithelia, the proximal face of the apical junctions are the sites of ncMTOC assembly, and MTs are organized along the planar axis with a proximal-distal polarity [304]. As pupal development proceeds, MTs become progressively more organized and are anchored not only at the proximal apical junction ncMTOC but also at the distal apical junction where their plus ends terminate (Figure 10C). The polarity of these MTs and their origin at the proximal face of the apical junction was established by live imaging of EB1-GFP comets [301,304,305]. The proximal–distal polarized array of MTs is required for the polarized transport of planar cell polarity (PCP) proteins [301,305]. Another ncMTOC transition occurs by very late pupal wing epithelia, when trichomes have formed and centrosomes are lost, multiple ncMTOCs assemble on the apical membrane and the MTs are organized along the apical basal axis [299,300] (Figure 10E).

During wing development, core PCP proteins such as Disheveled, Frizzled and Flamingo localize asymmetrically along the proximal-distal axis to coordinate planar-polarized development of the wing along the proximal-distal axis [306]. The polarized organization and dynamics of non-centrosomal MTs in wing epithelia is dependent upon the PCP pathway components Fat (Ft) and Dachsous (Ds), but these atypical cadherin proteins are not localized to the apical junctions where the MTOC resides [301,304]. Ft and Ds are regulated by Par-1 kinase, a key regulator of proximal-distal polarity in the developing wing [304]. Ectopic expression of Ds in the proximal compartment was sufficient to reverse MT polarity in wing epithelia and to change PCP [304]. Alternatively, cell shape or geometry appears sufficient to regulate MT organization independently of polarity mediators in embryonic epidermis as well as pupal wing epithelia [9].

Compared to the better-characterized apical–basal polarized MTs in other epithelia [254,307], very little is known about how this ncMTOC is assembled and regulated in the larval and pupal wing epithelia. The apical junction structure does not appear to be an adherens junction as it does not contain α- or β-catenins. Neither does it contain localized patronin or γ-tubulin and *patronin* RNAi knockdown shows no PCP phenotype [301]. Nin, on the other hand, is highly enriched at the site of MT assembly in larval wing disc epithelia [281]. Furthermore, because the majority of Nin is not associated with centrosomes in this cell type [281], Nin appears to be a unique structural component of this ncMTOC in the wing epithelia. Another possible component is the adhesion molecule Pio. Loss of Pio causes the absence of specialized MT bundles from pupal wings, suggesting that Pio plays a role in MT organization in the wing epithelia [295]. In *Drosophila* tracheal cells, Pio is required to anchor γ-tubulin to the ncMTOC site at the apical membrane [292]. It appears unlikely, however, that wing epithelial cells share the same ncMTOC regulatory mechanism since the wing ncMTOC is based on an intercellular junction that does not recruit γ-tubulin.

##### Eye: Photoreceptor and Cone Cells

**Figure 11:**
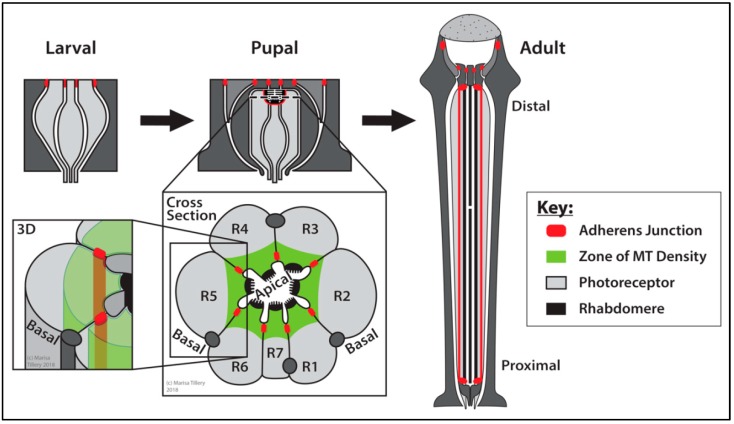
MTs are organized in photoreceptor cells near the apical membrane where the rhabdomere forms. This set of MTs appear to be organized from an undefined ncMTOC.

The *Drosophila* compound eye is comprised of approximately 800 ommatidia, the optical units of the eye. Each ommatidium is made of four cone cells, two primary pigment cells, and eight photoreceptor cells (R1-8) [308]. The photoreceptor cells are clustered in a seven-cell ring structure, and each extends a rhabdomere (long microvillar structures where rhodopsin and other signaling proteins reside) into the central core of the ommatidium (Figure 11). The R1-6 photoreceptors span nearly the entire length of the ommatidium (the length of the retina) while the R7 photoreceptor sits atop the R8 photoreceptor. During development, the adult compound eye forms from a monolayer epithelium within the eye-antennal imaginal disc.

At the third instar larval stage, photoreceptor cells undergo retinal cell fate determination to form eight-cell clusters in the eye-antennal imaginal disc. During this determination process, nuclei migrate from the basal side towards the apical side of imaginal eye disc cells. This nuclear migration is coordinated by the centrosome in conjunction with dynactin (activator of the MT-based motor dynein) and the Linker of Nucleoskeleton and Cytoskeleton (LINC) [309] complex [283,310,311]. As differentiation proceeds, Cnn and Spd-2 are lost from photoreceptor centrioles, and by pupal stages, most of the centrioles are also eliminated [54].

During the pupal stages, each photoreceptor cell elongates and the microvillar rhabdomere forms at the apical side (Figure 11). At mid-pupal development, the apical domain consists of an early rhabdomere structure and apical membrane. Each photoreceptor cell is connected to its neighbor by adherens junctions located on the adjacent basal side of the apical domains. At this stage, stabilized acetylated MTs are enriched at a subapical location (basal to the adherens junctions) of each photoreceptor cell (Figure 11). The location of the minus ends of these MTs and thus their polarity, however, has not been established [312].

The centrosomal proteins Cnn and γ-tubulin are localized just basal to the cluster of acetylated MTs at the distal region of mid-pupal rhabdomeres. However, they do not colocalize with the MTs and their role in organizing the MTOC adjacent to the adherens junction is unclear [313]. Interestingly, Cnn and γ-tubulin also exhibit perinuclear localization in mid-pupal photoreceptor cells, but whether this establishes a perinuclear MTOC has not been established [313]. Overexpression of *cnn* in photoreceptors using GMR-GAL4 (an eye driver) causes expansion of the apical domain, although the impact on MT organization has not been examined [313]. A *cnn* null mutant exhibits mild impairment in the placement of MTs and localization of apical membrane and adherens junctions proteins at the subapical layer and the apical domain, respectively [313]; however, *cnn* mutants display no overt disruption of ommatidia [88]. A genetic interaction between *cnn* and *baz* was discovered in a genetic screen which revealed that reduced expression of *cnn* enhanced the adult rough eye phenotype seen with *baz* overexpression [313]. This suggests that Cnn and Baz work together to contribute to ommatidium organization. However, the connection linking *cnn* to the presumed apical-distal ncMTOC is unclear.

In addition to Cnn, several other proteins may play a role in the presumed photoreceptor ncMTOC. Mutation in *shot* and *shot* overexpression results in severe disruption of the apical domain, the adherens junction, and MT organization [314]. The role of Patronin, perhaps implicit in the *shot* mutant phenotype, as well as the roles of other MT minus end regulators require further investigation in this system. The available data seem to indicate that the loss of centrosomes coincident with the generation of one or more ncMTOCs is critical for photoreceptor cell morphogenesis and function, although these ncMTOCs have yet to be clearly defined in this system.

*Drosophila* cone cells also experience a shift in MTOCs. During the larval and pupal stages of eye development, centrosomes are progressively lost from cone cells. These cells then assemble ncMTOCs during the pupal stage at the apical and basal plasma membranes where the MTs are aligned along the apical-basal axis [54,315]. Secretion from pupal cone cells is necessary to construct the lens and the pseudocone; however, further investigation is necessary to understand the role of this ncMTOC in the secretory function of the cell.

##### General Conclusions about Epithelial Cells

Overall, the organization of ncMTOCs in epithelial cells appears to be an essential and widespread phenomenon in *Drosophila* and other organisms. In their respective cell types, these ncMTOCs are involved in polarized processes, including transport, (e.g., Rab11 trafficking in follicle cells, chitin secretion in the trachea, and PCP in the wing) and also morphogenesis (e.g., the salivary gland and trachea). Epithelial cell ncMTOCs involve MT minus end regulators such as Patronin, γ-tubulin, and Nin located at the apical membranes or adherens junctions. Likely redundancies exist, as null mutation of *nin* has no overt phenotypes in *Drosophila*, and NOCA-1 (Nin ortholog) and Patronin cooperate in *C. elegans* epidermal cells [276]. In multiple cell types in *Drosophila*, Shot appears to anchor Patronin to stabilize MT minus ends, but in some cell types the related spectrins may also function to anchor Patronin. The roles of Shot and Patronin as key regulators of ncMTOCs in epithelial cells appears to be conserved in other species [3,316]. The involvement of these different MT minus end regulators in each of the epithelial cell types investigated so far remains to be fully determined to establish what common and divergent mechanisms are employed to organize functional MTOCs and fulfill the varied functions of each epithelial cell type.

#### 3.2.2. Non-Epithelial Cells

##### Oocyte: Transition from Centrosomal to ncMTOCs

**Figure 12:**
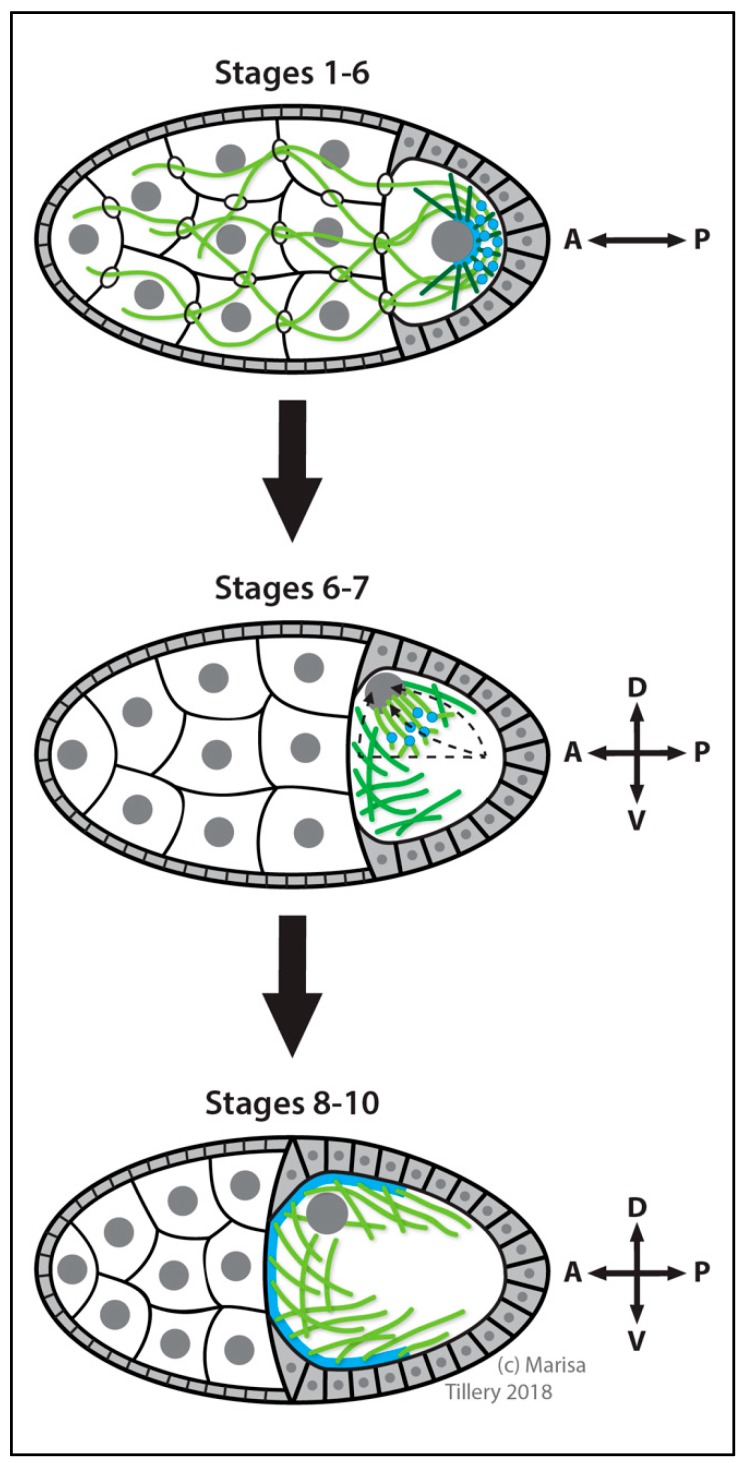
Dynamic changes in MTOCs during oocyte development. In stages 1–6, a cluster of centrioles at the oocyte posterior organizes an MTOC together with a ncMTOC assembled on the posterior hemisphere of the oocyte nucleus. At stage 7, the oocyte migrates to the anterior-dorsal corner of the oocyte and then a new ncMTOC assembles on the anterior cortex. Nuclei are darker grey; MTs are green; centrioles (dots) and other MTOCs are blue.

During oogenesis, the oocyte develops in an egg chamber consisting of one oocyte at the posterior end and 15 nurse cells interconnected by ring canals that permit intercellular trafficking (Figure 12, Stages 1–6). Early in oogenesis, the oocyte is specified from among the fifteen other cells that become nurse cells. One feature of oocyte specification is MTOC establishment: MT minus ends are focused in the oocyte with the MT array extending through the ring canals into all fifteen nurse cells. Disruption of the MT array via colchicine treatment causes loss of oocyte specification and oocyte-specific mRNA/protein localization [317,318]. Moreover, the loss of Dynein motor complex components Bicaudal-D (BicD) or Egalitarian (Egl) disrupts the localization of oocyte-specific mRNAs/proteins, oocyte specification, and MT polarization [319,320,321,322,323,324]. Thus, a major role for the posterior oocyte MTOC is to organize the MTs necessary for the transport of essential oocyte-specifying mRNAs and proteins by a dynein-BicD-Egl complex. 

A key event in oocyte specification is the migration of the centrioles of the nurse cells to the oocyte. The centrioles then cluster at the posterior end of the oocyte to form the MTOC in stages 1–6 egg chambers (Figure 12, Stages 1–6). However, unlike the transport of mRNA/protein, centriole migration might not be reliant on the dynamic MT array as colchicine treatment does not disrupt their transport [319]. Centriole migration is, however, reliant on dynein and Shot as loss of either disrupts the localization of centrioles to the oocyte [319,325]. The reliance on dynein seems to contradict the lack of reliance on MTs for centriole migration; however, the effect of dynein disruption on centriole transport might be indirect [325,326]. The mechanisms of centriole transport to the oocyte and the relative contributions of MTs and actin require further elucidation. Overall, the transport of the egg chamber centrioles to the oocyte posterior and the establishment of the MTOC there is critical for oocyte development.

##### A Centrosomal MTOC Cooperates with a Nuclear ncMTOC in Oocyte Nuclear Migration

The cluster of centrioles that forms the MTOC in stages 1–6 egg chambers is localized to the posterior end of the egg chamber in-between the nucleus and posterior follicle cells (Figure 12, Stages 1–6). These centrioles are inactive during migration as they cannot nucleate MTs [319,327]. Until the centrioles arrive, MTs are dispersed in the cytoplasm of the oocyte. However, when the centrioles reach the posterior end of the oocyte, the centriole cluster behaves as one large MTOC that recruits PCM components including γ-tubulin, Plp, Cnn, and TACC [98,175,328]. The centriole cluster is not necessary for oogenesis to proceed [178], however, suggesting that there are alternative modes of MTOC organization in the oocyte.

In addition to the centriole cluster at the oocyte posterior, a ncMTOC assembles on the posterior hemisphere of the oocyte nucleus [329] (Figure 11, Stages 1–6). MT regrowth assays reveal that MT nucleation is asymmetrically enriched at the posterior hemisphere of the nucleus next to the centrosomal MTOC [329,330]. Both isoforms of γ-tubulin localize to the hemi-perinuclear ncMTOC [329] along with Mud [163,330], Asp [330], Calmodulin (Cam) [331], and Dynein light intermediate chain (Dlic) [331].

During stages 6–7, this hemi-perinuclear ncMTOC supports nuclear migration [332] when the nucleus, along with the cluster of centrioles, migrates from the posterior to the anterior end of the oocyte. The posterior MTOC provides a pushing force that moves the nucleus along the anterior membrane while the hemi-perinuclear ncMTOC pushes the nucleus along a lateral membrane path [329,330,331,332] (Figure 11, Stages 6–7). Further support for the MTs involvement in pushing forces comes from MT laser ablation experiments [330,331]. Mud, whose localization to the nuclear ncMTOC depends on Asp, is required for the perinuclear ncMTOC and the lateral membrane pushing force. The posterior MTOC, on the other hand, relies on centrioles as it is impaired in a *sas-4* mutant that blocks the anterior membrane pushing force. These two MTOC activities redundantly move the nucleus to its dorsal-anterior position in the oocyte as eliminating both MTOC activities blocks nuclear migration in about 50% of oocytes [330,332]. 

The signal that causes the MT rearrangement and initiates migration is reliant on Gurken (Grk) and comes from the posterior follicle cells [331,332,333]. This is evident in *grk* mutant oocytes where polarity is disrupted and the nucleus does not migrate [333,334]. Nuclear repositioning at the anterior-dorsal wall, together with the recruitment of Grk mRNA and protein there, establishes the position of the anterior-dorsal coordinate of the embryo [333,334,335,336,337].

##### A Repolarization of MTs Creates an Anterior ncMTOC

Once the nucleus migrates to the anterior cortex of the oocyte, the minus ends of the MTs are no longer located posteriorly; rather, they are located at the anterior membrane, establishing an anterior cortical ncMTOC (Figure 12, Stages 8–10). This ncMTOC is organized by Shot, which is anchored to the anterior membrane via Shot’s actin-binding domain. Shot is excluded from the posterior membrane by Par-1 via an unknown mechanism. Both Shot and Par-1 are required for the anterior localization of the ncMTOC as mutant oocytes of either cause MTs to disperse throughout the oocyte. MT regrowth experiments using colcemid combined with its ultraviolet (UV) inactivation demonstrate that MTs regrow from Patronin foci. Shot recruits Patronin to generate this ncMTOC through a mechanism that does not require γ-tubulin or PCM proteins. Instead, it has been hypothesized that Shot/Patronin captures and stabilizes MT minus ends to seed new MT growth with the aid of katanin [271].

As the oocyte matures, the centrioles are gradually degraded by a mechanism that involves the loss of Polo and the PCM that maintains centriole stability. As centrioles are detected in egg chambers up to stage 13, it is proposed that they progressively decrease in number from stages 10-13, correlating with a decrease in Polo activity [338]. Oocytes are not the only tissues that lose centrioles; polyploid tissues in flies generally lack centrioles [54,339,340], but how this is regulated remains unclear.

Overall, the MTOCs in the oocyte are very dynamic in their locations and their structural composition. At around stage 6, a posterior MTOC comprised of a cluster of many centrioles and centrosomal PCM proteins functions with a perinuclear hemispheric ncMTOC comprised of Asp and Mud. By stage 10, following nuclear migration, the MT polarity shifts 180° and the dominant MTOC is an anterior cortical membrane ncMTOC comprised of Shot and Patronin. These MTOCs in the developing oocyte are essential for establishing the anterior-posterior and dorsoventral axes for the future embryo through nuclear migration and polarized protein and mRNA localizations [175,341,342].

##### Muscle

**Figure 13:**
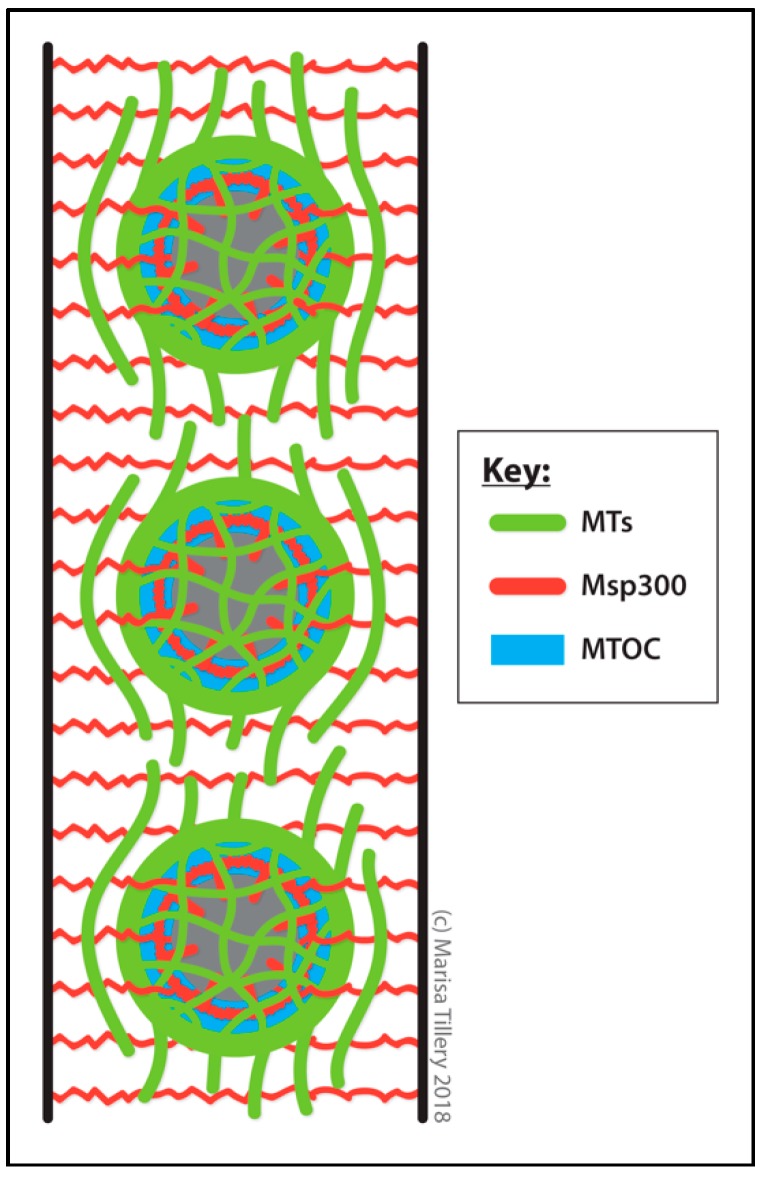
Perinuclear ncMTOC in muscle cells. The regular spacing of nuclei in multinucleate muscle cells requires the perinuclear ncMTOC.

The differentiation of myoblasts into myotubes is a critical process during muscle development accompanied by significant rearrangements of the MT cytoskeleton. MT organization during myogenesis has been well-characterized in in vitro vertebrate myoblast cultures [275,343]; however, relatively little is known about how this process is regulated in vivo. Cultured myoblasts have a centrosomal MTOC, but during differentiation into myotubes, PCM proteins gradually relocate from the centrosome to the nuclear periphery to establish a perinuclear ncMTOC [275,343]. Consistent with this in vitro MT reorganization, the MT network is also developmentally regulated in vivo in the *Drosophila* muscle. Although it is unclear at which stage centrosomes are lost during muscle development, *Drosophila* larval muscles lack centrosomes and MTs are organized at non-centrosomal sites, particularly at the nuclear periphery (Figure 13). Myotubes in the late embryo (stage 16) exhibit a polarized MT organization with MT plus ends close to the myotendinous junction [289]. Following muscle maturation, the MTs in larval striated muscle undergo a dramatic reorganization, becoming enriched at perinuclear sites and radiating outward from the nuclear periphery.

Some proteins that comprise or regulate the muscle perinuclear ncMTOC have been examined to date, yet it is unclear whether the nuclear surface represents a nucleation site in vivo as no MT regrowth experiments or EB1 live imaging experiments have been reported. Gamma-tubulin shows diffuse cytoplasmic staining in mononucleated myoblasts but then accumulates at discrete punctae at the nuclear periphery upon myoblast fusion into multinucleated myoblasts [344]. In addition, the MT anchor Nin is present at the nuclear surface in *Drosophila* larval muscle [281]. Thus, muscle myotubes appear to assemble a perinuclear ncMTOC.

The *Drosophila* perinuclear muscle ncMTOC is important for normal muscle physiology, but its functions are poorly understood. A hallmark of myotubes is the evenly-spaced distribution of most nuclei at the periphery of the myofiber (the exception being nuclei in the myofibers of specialized regions such as the neuromuscular junction) [345,346]. MT-associated proteins such as Ensconsin (Ens), Shot, and EB1 and the MT-motor proteins dynein and kinesin play critical roles in maintaining proper nuclear positioning in *Drosophila* embryonic and larval muscle [347,348,349]. Furthermore, overexpression of *spas* in *Drosophila* second-instar larval muscle disrupts myonuclear positioning, demonstrating a role for intact MTs in myonuclear positioning [350].

The LINC complex controls nuclear positioning in a variety of cell types in *Drosophila* and other organisms [309,351,352]. In the LINC complex, Klarsicht, ANC-1, Syne Homology (KASH) domain proteins (Nesprins) transverse the outer nuclear membrane and connect the nucleus to cytoskeletal structures and bind to Sad1p, UNC-84 (SUN) domain proteins that span the inner nuclear membrane and connect to the nuclear matrix and chromatin. In *Drosophila* muscle, organization of the perinuclear MT network requires the cooperative action of Muscle-specific protein 300 kDa (Msp300) and Klarsicht (Klar), two KASH-domain proteins [353]. Msp300 assembles into filaments that traverse the nuclear membrane, forms a supra-perinuclear ring and is also incorporated into the Z-discs [353] (Figure 13). Recent studies indicate that Nesprins associate with centrosomal proteins for MT organization in vertebrate myotubes. Centrosomal proteins including Akap450, PCM1, Pericentrin, CDK5RAP2, and γ-tubulin were reported to localize to the nuclear surface during vertebrate skeletal muscle formation [275,354,355]. In mouse myoblast culture, Nesprin-1 is essential for perinuclear recruitment of Pericentrin, PCM1, γ-tubulin, Akap450 and other PCM proteins [356]. Akap450, but not Pericentrin or PCM1, is required to nucleate MTs at the nuclear envelope and to ensure proper myonuclear positioning [357]. Whether similar mechanisms exist in *Drosophila* muscle remains to be shown.

While investigation of the *Drosophila* muscle has advanced our understanding of the structure and function of the perinuclear ncMTOC, additional insight is available from studies of mammalian muscle and the functions of MTs during muscle differentiation and maturation [345,358]. Importantly, defective positioning of myonuclei has been observed in a variety of muscular diseases including Duchenne muscular dystrophy, Becker muscular dystrophy, and Emery-Dreifuss muscular dystrophy [345,346], suggesting that maintaining nuclear positioning is essential for preventing muscular degeneration.

##### Neuron

**Figure 14:**
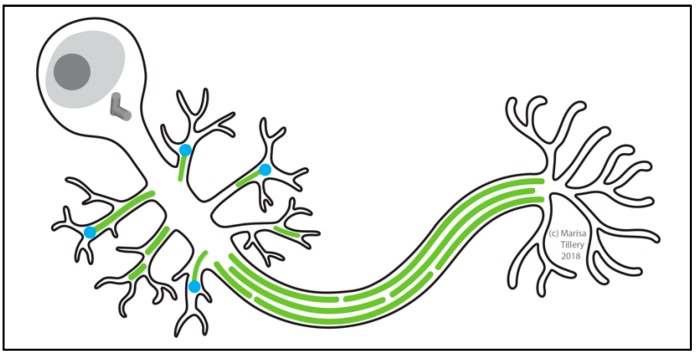
Neurons assemble ncMTOCs within dendrite branches. Neuronal ncMTOCs regulate dendrite branching. The nucleus is dark grey; MTs are green; ncMTOCs are blue.

In *Drosophila* neurons, axonal MT plus ends are directed away from the cell body. In contrast, ~90% of dendritic MTs are oriented with their plus ends pointing towards the cell body where the centrosome resides, indicating that these MTs are not organized by the centrosome [359,360] (Figure 14). To further support this, centrosome removal either by laser ablation or mutation of *sas-4* in larval sensory neurons disrupts neither neuronal MT organization nor polarity [361]. Currently, the composition of the sites of MT nucleation in neurons remains unclear.

Cnn is present at dendritic ncMTOCs. It appears to be a transcriptional target of *abrupt*, and it represses dendrite branching particularly in class IV dendritic arborization (da) neurons where *cnn* and *plp* mutants display more elaborate dendrites [362]. Other work has shown through live cell imaging analysis that EB1-GFP comets (indicative of growing MT plus ends) emanate from sites in dendrites that frequently coincide with Golgi outposts. These sites contain γ-tubulin and Plp and require γ-Tub23C and Plp function for normal dendrite branch extension and stability [363]. Golgi are well-established as sites of cellular ncMTOC generation [364,365,366], but it is not yet clear if this is the case in *Drosophila* and it remains controversial whether Golgi outposts are the sites of ncMTOCs in *Drosophila* neurons. Gamma-tubulin and Golgi outposts do not co-localize extensively in dendrites, most notably in class I da neurons where Golgi are not found at dendritic branch points where γ-tubulin localizes [367]. Additionally, forcibly dragging the Golgi out of dendrites changes neither the localization nor the concentration of γ-tubulin at dendritic branches [367], arguing that Golgi outposts may not be the nucleation sites for non-centrosomal MTs in dendrites. Further investigation is necessary to understand the subcellular structures that comprise the ncMTOCs in neuron dendrites.

##### Spermatid

**Figure 15:**
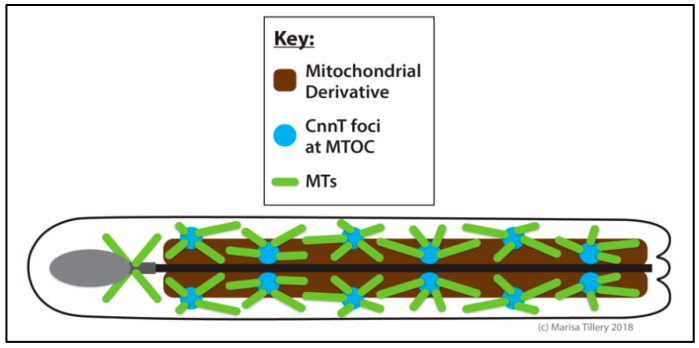
A splice variant of Cnn assembles ncMTOCs on the surface of spermatid mitochondria. During spermatid differentiation and elongation, CnnT localizes to mitochondrial derivatives, recruits γ-TuRCs, and converts mitochondria to ncMTOCs.

During spermiogenesis, mitochondria in the newly-formed spermatid fuse to form two giant mitochondrial derivatives that wrap together into a spherical structure called the nebenkern. During spermatid elongation, the two mitochondrial derivatives unfurl and elongate along the full length of the spermatid tail [368,369,370]. MTs are essential for spermatid elongation and the mitochondria serve as a platform upon which ncMTOCs form [92,371] (Figure 15).

Alternative splicing of Cnn produces a testis-specific splice variant called CnnT that retains the CM1 domain but lacks the centrosome targeting domain at its C-terminus, instead expressing a unique C-terminal mitochondrial-targeting domain. CnnT localizes to the mitochondrial derivative and there recruits and activates the γ-TuRC via its CM1 domain to promote MT nucleation at this unique ncMTOC [92]. CnnT assembles into foci on the mitochondrial surface (Figure 15) but does not appear to recruit additional PCM proteins besides the γ-TuRC. An engineered protein consisting of just the CM1 domain from either Cnn or vertebrate CDK5RAP2 fused to the CnnT mitochondrial-targeting domain was sufficient to convert mitochondria to MTOCs [92]. This MTOC is unique in both its strength and its molecular simplicity. It also demonstrates a design principle in MTOC generation: spatial targeting of the CM1 domain is sufficient to generate localized MTOCs.

### 3.3. Summary of ncMTOCs

Active research in this field is beginning to yield answers, yet we continue to have little understanding of how centrosomes are inactivated, how MTs are remodeled, and how the variety of ncMTOCs form in most cell types where they have been documented. ncMTOCs are diverse in their subcellular locations, architecture, and how they regulate MT assembly. γ-tubulin is a major regulator of MT assembly at centrosomes and is required for CnnT to generate ncMTOCs at mitochondria and for the ncMTOCs in trachea and neurons but was not detected at several other ncMTOCs, suggesting that alternative minus end regulators are involved. Whether γ-tubulin is truly dispensable at other ncMTOCs has not been rigorously tested. As investigations of *Drosophila* ncMTOCs advance, we expect additional examples of ncMTOCs that form independent of γ-tubulin and centrosomal proteins to be discovered. An emerging key MT regulator at ncMTOCs is Patronin, which is essential at the anterior oocyte and the follicle cell apical membrane ncMTOCs. In both instances, the spectroplakin Shot anchors Patronin at the cell membrane. Patronin and Shot likely function at other ncMTOCs as well. Patronin and γ-tubulin are probably not the only MT regulators at ncMTOCs, and it is likely that others such as Nin will emerge as the field advances. Additionally, the diversity of ncMTOCs must require site-specific proteins to tether MT organizing proteins. Shot and β-spectrin anchor Patronin in oocytes and follicle cells, and Pio is required for γ-TuRC recruitment in the trachea. However, little is known regarding site-specific tethers employed at other ncMTOCs. More research is needed to better characterize the proteins that compose, anchor, and regulate the diverse ncMTOCs in *Drosophila*.

## 4. Conclusions

*Drosophila* is a powerful model organism, enabling investigations into the functions of centrosomes and ncMTOCs, their dynamic changes, protein compositions, regulation of their assembly, and their functions in cells and for development in a broad range of tissue and developmental contexts. The centrosome is clearly essential in several cell types and developmental stages in *Drosophila* (e.g., embryonic cleavage cycles, male meiosis, wing epithelial divisions, and the assembly of cilia in sensory neurons) despite the fact that flies can develop into fully formed adults without centrosomes. Evidently, backup mechanisms exist to support acentrosomal mitosis, enabling mitotic spindle MT assembly in part by utilizing mechanisms in place for acentrosomal female meiotic spindle assembly. In many cell types, ncMTOCs play critical roles for a variety of cell type-specific functions. The diversity of these ncMTOCs in terms of their compositions, subcellular localizations, mechanisms of MT assembly, and the biological functions they support are beginning to come into focus.
